# Stage-Adapted InSAR–Probability Integral Method Fusion for Monitoring and Spatially Continuous Reconstruction of Repeated-Mining Subsidence

**DOI:** 10.3390/s26216706

**Published:** 2026-10-23

**Authors:** Miaomiao Ma, Youfeng Zou, Huabin Chai, Lailiang Cai, Chunhui Chen, Weiwei Zhou

**Affiliations:** 1 School of Geomatics and Aerospace Information, Henan Polytechnic University, Jiaozuo 454000, China; mamiaomiao@home.hpu.edu.cn (M.M.);; 2 School of Surveying and Mapping Engineering, Henan College of Surveying and Mapping, Zhengzhou 451464, China; 3 Henan Geophysical Spatial Information Research Institute Co., Ltd., Zhengzhou 450009, China

**Keywords:** mining subsidence, repeated mining, low-hilly terrain, InSAR, probability integral method, data fusion

## Abstract

**Highlights:**

**What are the main findings?**

- Stage-adapted D-InSAR and SBAS-InSAR processing yielded configuration-specific empirical effective retrieval limits of approximately 149 and 194 mm, respectively.
- In the same-site spatial-block assessment, fusion increased RMSE by 1.25% relative to PIM during initial mining and reduced it by 3.62% during repeated mining. These post-selection results do not represent external validation.

**What are the implications of the main findings?**

- The InSAR–PIM framework retains observations at valid basin margins and supplies model estimates where central InSAR observations are unavailable.
- Magnitude-driven weighting removes the theoretical value jump at the magnitude thresholds and reduces local gradients relative to hard-threshold and fixed-weight benchmarks.

**Abstract:**

Repeated-mining subsidence in low-hilly terrain causes InSAR decorrelation and incomplete deformation fields. At the 2301 working face in Shanxi Province, China, this study combines InSAR observations near basin margins with central estimates from the probability integral method (PIM). *D*-InSAR and SBAS-InSAR were applied to the initial-mining and repeated-mining stages, with operational retrieval limits of 149 and 194 mm. PIM was fitted to three late field campaigns per stage, with development factors constrained by the complete observation sequences. Fusion weights varied with PIM-predicted subsidence magnitude. Full-data fusion RMSEs of 28.58–134.78 mm (R^2^ = 0.956–0.990) describe internal agreement. Same-site spatial-block fusion RMSEs were 62.16 and 166.79 mm for the two stages, respectively: 1.25% higher and 3.62% lower than PIM, and 81.3% and 76.6% lower than InSAR alone, largely reflecting its underestimation of much larger central subsidence. The same folds informed model selection, so these are not external validation results. Symmetric power-ratio weighting removed theoretical threshold jumps and reduced local transition gradients relative to hard-threshold and fixed-weight fusion. Sparse transition observations limited assessment of predictive accuracy. The supported benefit is retention of margin observations and model-based completion of central gaps with a continuous threshold transition, rather than consistent accuracy improvement over PIM.

## 1. Introduction

As a major primary energy source in China, coal plays an important role in national energy security and economic development [1]. Shanxi, a core coal-producing base in China, has most of its mining areas distributed across the topographically complex Loess Plateau and low-hilly regions. Pronounced terrain relief, thin unconsolidated layers, and thick bedrock alter the mining-subsidence response in these areas [2]. Compared with the subsidence in plains, deformation is more asymmetric and nonlinear. Repeated mining intensifies cumulative surface subsidence, causing severe damage to buildings, pipelines, and other infrastructure. It may also trigger slope instability, ground fissures, and other geohazards, thereby threatening local ecosystems and the safety of residents [3]. Reliable long-term subsidence monitoring is therefore needed in low-hilly areas affected by repeated mining.

Surface deformation in mining areas is commonly monitored using ground-based geodetic techniques and remote-sensing methods. Conventional contact-based methods (e.g., precise leveling, static GNSS measurement, and total stations) can acquire millimeter-level, high-precision point data, but they are labor-intensive and offer limited spatial coverage [4], making it difficult to meet the demand for large-scale, continuous monitoring of the subsidence field. Interferometric synthetic aperture radar (InSAR) offers advantages such as all-weather capability, wide coverage, and high spatial resolution; since Berardino et al. proposed the SBAS-InSAR method [5], it has become an important tool for mining subsidence monitoring. However, mining-induced subsidence is typically accompanied by large deformation gradients and strong temporal decorrelation, and coherence is severely lost particularly in the high-gradient area at the center of the subsidence basin, leading to a loss of deformation information in the central zone [6,7]. This problem is even more prominent in low-hilly areas owing to the combined influence of vegetation cover and terrain relief [8].

To compensate for the loss of central information in InSAR, previous studies have combined D-InSAR and SBAS-InSAR with the probability integral method (PIM) for subsidence prediction, parameter inversion, and deformation reconstruction [9,10]. Gao et al. analyzed the joint application of SBAS-InSAR and PIM [11]. They described complementary roles for the two methods: InSAR maintains good coherence at the basin margin but decorrelates at the center, whereas PIM can compensate for the missing center but converges too rapidly at the basin margin. However, many existing multi-source fusion methods rely on hard-threshold stitching or fixed-weight fusion and lack mechanisms to ensure spatial continuity [12]. Differences in spatial resolution and coverage make smooth transitions difficult to achieve with simple zonal stitching [13]. Fusion boundaries may then contain gradient discontinuities or step-like artifacts that reduce reconstruction accuracy [14]. Although the recently improved 2D SBAS-InSAR method reduces basin margin measurement errors, it still does not resolve the dynamic weighting and smooth-transition problem in the fusion process [15].

Overall, existing InSAR–PIM joint reconstruction studies still exhibit three limitations. First, most studies focus on single-stage mining or relatively flat terrain, with insufficient attention to stage-adapted reconstruction under repeated mining in low-hilly terrain [16]. Second, profile leveling gives strong magnitude control but limited areal sampling; spatially distributed high-coherence InSAR observations near the basin margins can provide additional two-dimensional evidence, although their contribution must be quantified. Third, many fusion implementations lack an endpoint-matched transition rule, so hard source switching can introduce gradient jumps and a step effect.

This study examines the repeated-mining process of the 2301 working face in a low-hilly mining area of Shanxi Province. The analysis places the initial-mining stage (2301-1) and repeated-mining stage (2301-2) within a single InSAR–PIM framework to reconstruct subsidence and examine its spatial evolution. The main contributions are as follows:

(1) A stage-adapted InSAR processing framework was developed, in which D-InSAR was applied to the initial-mining stage with limited image availability, whereas SBAS-InSAR was applied to the repeated-mining stage with a denser image sequence. Under the adopted image and processing configurations, the empirical effective retrieval limits were approximately 149 mm and 194 mm, respectively.
(2) The framework is evaluated for the two mining stages of the 2301 working face using stage-specific InSAR products and field observations. The primary PIM inversion uses only field observations from three late campaigns. A secondary inversion adds 15 systematically selected InSAR perimeter samples per stage to quantify their incremental effect. The evaluation distinguishes full-data agreement, spatial-block prediction, and transition continuity.
(3) A deformation-magnitude-driven symmetric power-ratio weighting mechanism was developed to provide a gradual transition from the InSAR-dominant basin margins to the PIM-dominant central decorrelation zone, thereby connecting margin observations to central model estimates in both mining stages. Here, dynamic refers to variation with predicted deformation magnitude, not to a separately estimated time-adaptive weighting model. The exponent *p* = 2 is retained as a moderate nonlinear setting, rather than an empirically optimized value.

## 2. Materials and Methods

### 2.1. Study Area

**Figure 1:**
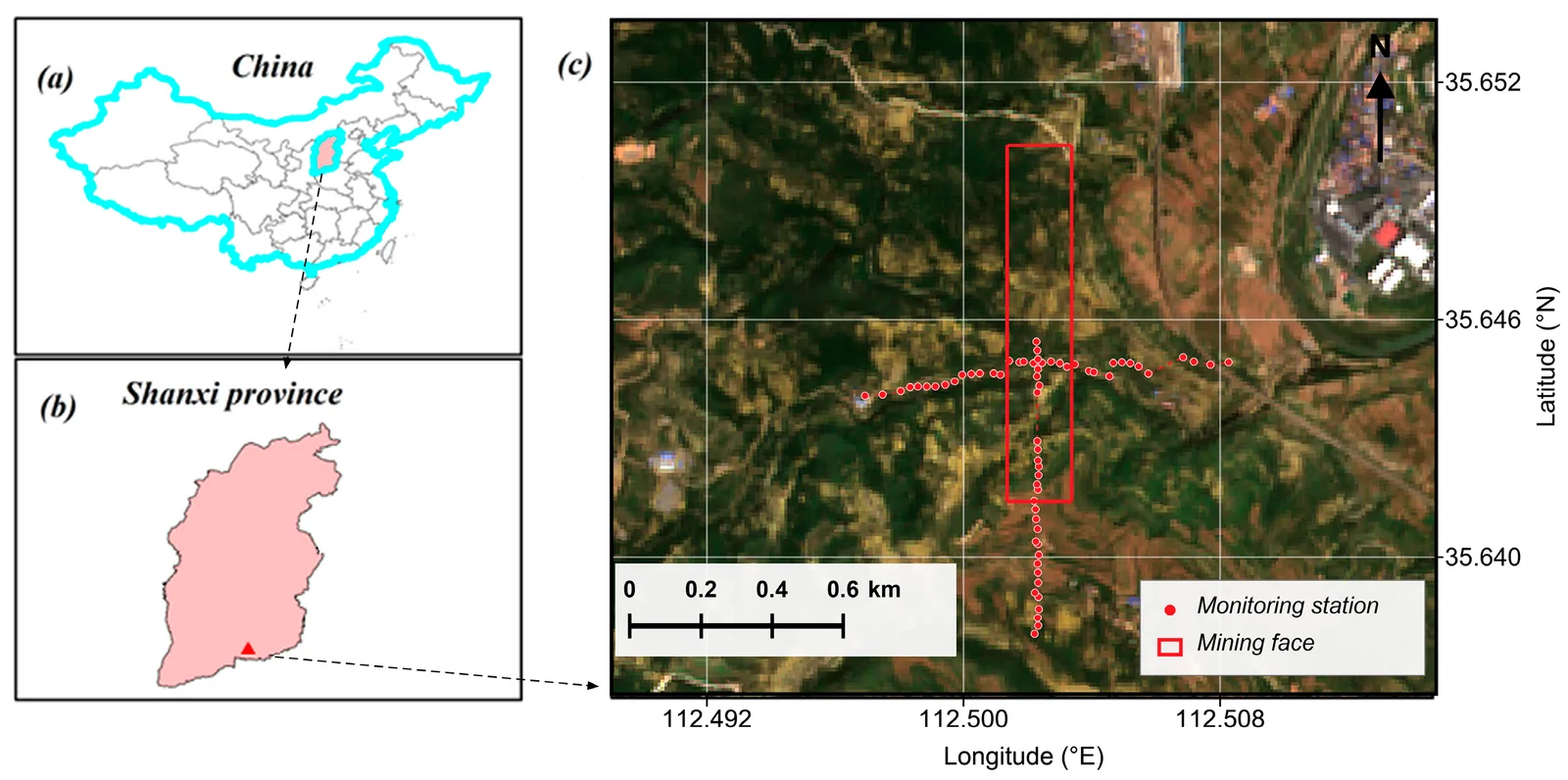
Study area. (**a**) Location of Shanxi Province in China. (**b**) Location of the study area in Shanxi Province. (**c**) Detailed layout of the 2301 working face and monitoring lines on a Sentinel-2A. Level-2A true-color image acquired on 3 September 2018 (contains modified Copernicus Sentinel data [2018]); coordinates are WGS84.

The study area is located on the southern margin of the Qinshui Basin and on the western side of the southern end of the Taihang Mountains, representing a typical low-hilly mining area. The regional landforms are dominated by erosional mountains, with hilltops mainly covered by loess and bedrock exposed within the gullies, resulting in pronounced terrain relief. The highest point, at Keshan Temple in the west, is 877 m above sea level, whereas the Qinhe riverbed in the south-central area is lowest at 550 m. The elevation difference is 327 m, and the ground above the 2301 working face ranges from 560 to 710 m (Figure 1).

Mining activities in the study area primarily target the No. 3 coal seam of the Lower Permian Shanxi Formation. The coal seam is relatively stable, with an average dip angle of approximately 5° (2–7°), a full-seam thickness of 4.8–5.6 m (approximately 5.2 m on average), an unconsolidated surface layer thickness of approximately 20 m, and an average mining depth of approximately 390 m. The working face was extracted using fully mechanized strike-longwall mining with full caving.

**Figure 2:**
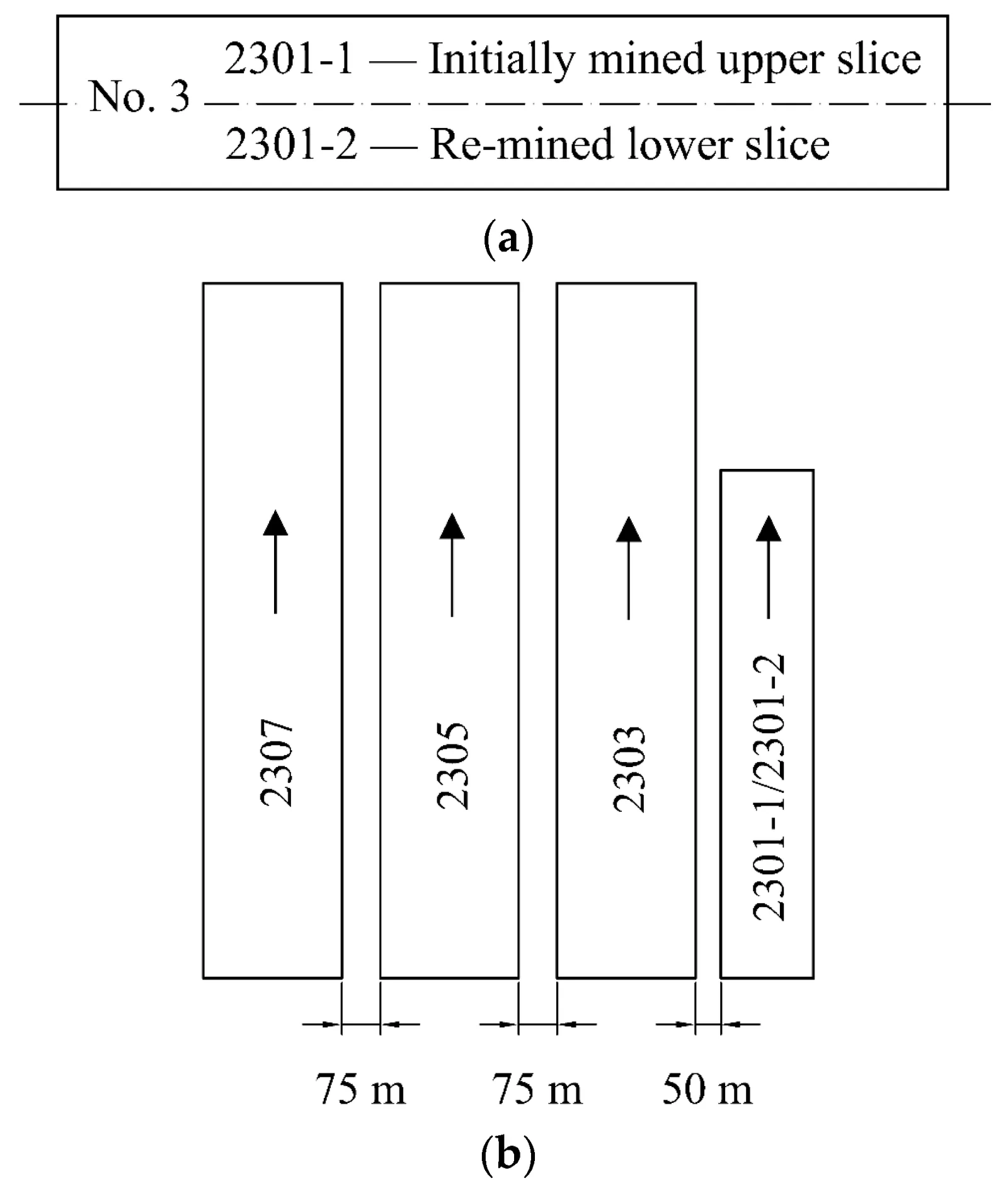
Layout of the repeated-mining working face (2301). (**a**) Positions of the 2301-1 (**upper slice**) and 2301-2 (**lower slice**) in the No. 3 coal seam. (**b**) Plan view of adjacent working faces, with arrows indicating the advance direction.

The analysis focuses on the repeated-mining process of the 2301 working face (Figure 2). The 2301 working face was mined in slices: 2301-1 represents the initial-mining stage (upper slice), with a mining thickness of 2.6 m, a dip length of 176 m, a strike length of 996 m, an average mining depth of 390 m, and a mining period from May 2015 to January 2016. The 2301-2 stage represents the repeated-mining stage (lower slice), with a mining thickness of 2.4 m, a dip length of 158 m, a strike length of 1021 m, an average mining depth of 390 m, and a mining period from July 2018 to January 2019. The detailed mining parameters are summarized in Table 1. These stages cover the repeated-mining sequence examined here.

The adjacent working faces 2303, 2305, and 2307 are sequentially located west of 2301 (Figure 2b). The nearest working face, 2303, is separated from 2301 by a protective coal pillar of approximately 50 m and was mined from May 2016 to May 2017 (Table 1). Unlike 2301, 2303 was mined through the full seam thickness of 5.2 m without slicing. Before the extraction of 2301-2, the overburden had been affected by upper-slice extraction at 2301-1 and may also have experienced lateral disturbance from 2303. These prior workings form part of the preconditioned overburden setting; their inherited structural influence should be distinguished from displacement accumulated during the monitoring interval.

Extraction of 2303 ended in May 2017, before SBAS-InSAR monitoring began in June 2018; this timing alone does not establish that residual subsidence had ended. Although the extraction of the 2305 working face partially overlapped with the early monitoring period, both 2305 and 2307 were farther west. Their spatial separation alone does not provide a quantitative bound on their contribution. The absence of a separately identifiable basin does not exclude a local contribution to the measured 2301-2 flanks. Any post-June-2018 compaction from older goafs is included in the observed cumulative field and cannot be separated from deformation induced by 2301-2 using the available observations. In this study, “inherited damage” therefore denotes the preconditioned overburden state, whereas “contemporaneous deformation” denotes displacement accumulated during the 2018–2019 observation interval.

Although the dimensions and mining period of 2303 are known, no dedicated post-mining monitoring or stability records were available to constrain its residual displacement during 2018–2019. A static PIM footprint represents spatial influence, not the remaining increment over that interval. The contribution of 2303 is therefore treated as a possible source of local asymmetry, not as negligible or independently quantified.

### 2.2. Data Sources

#### 2.2.1. Sentinel-1A SAR Images

Sentinel-1A synthetic aperture radar (SAR) imagery provided by the European Space Agency (ESA) was the primary data source for surface deformation monitoring. The selected data are single-look complex (SLC) products acquired in VV polarization and interferometric wide-swath (IW) mode, with a revisit period of 12 days.

For the 2301-1 initial-mining stage, the archived same-track ascending source set contained 13 Sentinel-1A SLC scenes acquired from July 2015 to May 2016. The final cumulative D-InSAR chain used 12 of these scenes to form 11 sequential interferometric pairs (Table 2). Although the source archive also contains an SLC dated 29 April 2016, no interferogram involving that scene was retained in the processing project, so it did not enter the cumulative chain. Pair selection therefore follows the interferograms retained in the archived processing project rather than an ideal 12-day sampling interval. Nine pairs had temporal baselines of 24 days, while two had baselines of 48 days. For the 2301-2 repeated-mining stage, 38 long-time-series Sentinel-1A SAR scenes acquired from June 2018 to September 2019 were selected (Table 3). Sentinel-1A data provide stable revisit intervals and all-weather monitoring capabilities, making them suitable for long-term deformation monitoring in mining areas [17,18].

#### 2.2.2. Ground Surface Movement Observations

**Figure 3:**
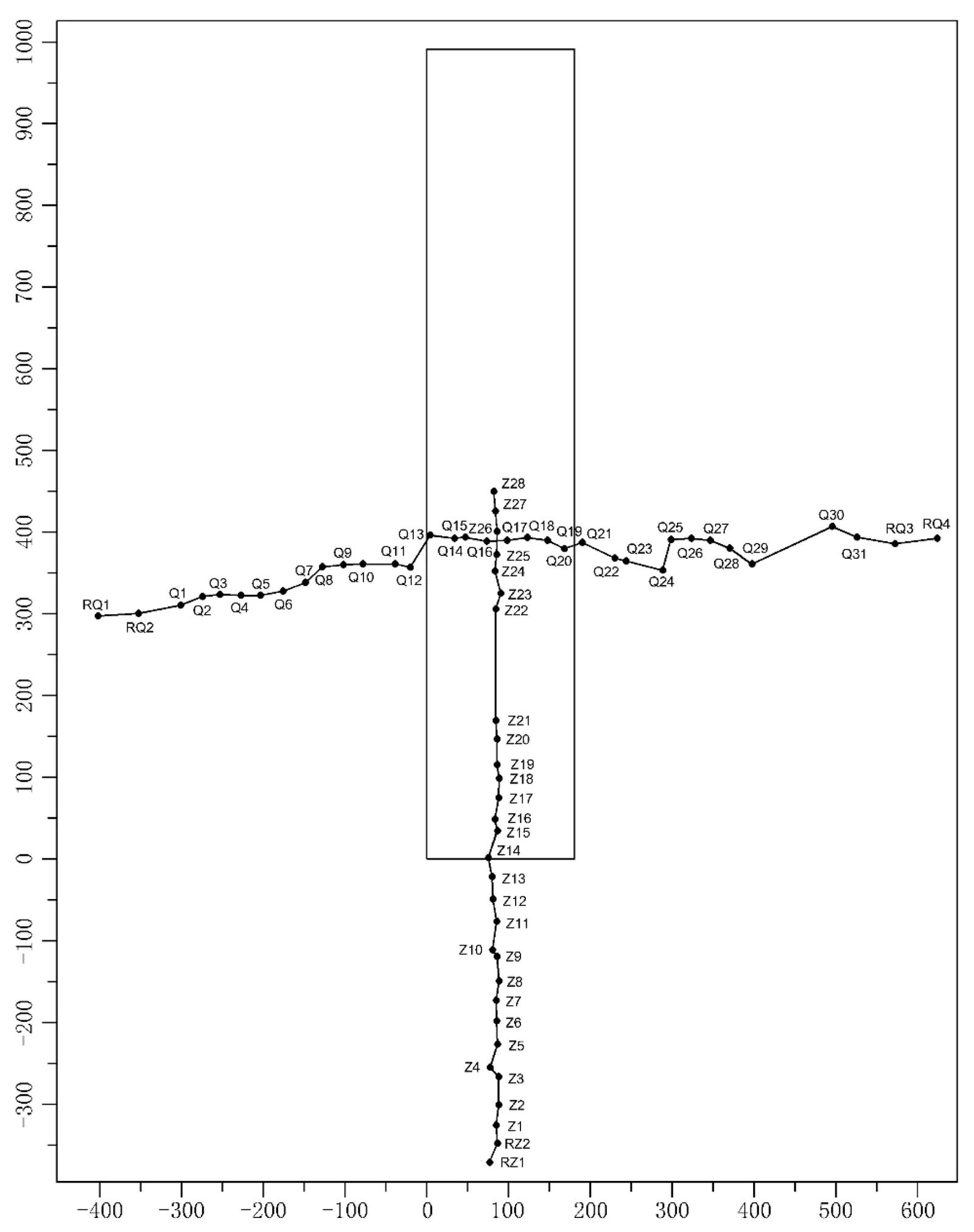
Layout of the surface movement observation network. The two principal profiles cross the InSAR-retaining basin margins, the steep transition flanks, and the central decorrelation zone; the point counts retained at the representative epochs were 30/35 (strike/dip) for 2301-1 and 28/34 for 2301-2.

A surface movement network was established on the eastern side of the mine field to measure mining-induced displacement (Figure 3). Its strike and dip observation lines were approximately 820 and 1026 m long and originally comprised 75 monuments in total. The retained analysis points are specified below.

The field observations were compiled from two non-public internal rock-movement monitoring reports, one for each working face (2301-1 and 2301-2). The reports specify leveling on flatter ground and RTK observations on steeper slopes; leveling accuracy was required to meet at least third-order standards. The retained 2301-1 profiles contain 24 leveling and 41 RTK points, and the 2301-2 profiles contain 22 leveling and 40 RTK points. Pointwise precision records were not available for defensible variance weighting. Vertical changes were reconstructed from archived heights relative to the supplied baselines. The stage-1 baseline is documented as the average of the 17 April and 3 May 2015 initial surveys; their archived baseline columns are identical, so the two original surveys cannot be checked separately. The sequences contain 21 and 12 post-baseline campaigns, respectively. Two stage-1 records marked as destroyed were excluded, leaving 1363 and 744 valid point-epoch observations. The representative profiles retain 30/35 and 28/34 strike/dip points. For 2301-1, the 16 July 2015 start state was interpolated between 13 and 28 July and subtracted from 28 May 2016, five days after the *D*-InSAR endpoint. For 2301-2, the 21 February 2019 endpoint is four days before the SBAS field; its 6 July 2018 baseline is six days after the SBAS zero.

#### 2.2.3. Auxiliary Data

During data processing, several auxiliary datasets were employed to support deformation monitoring and InSAR processing. A 30 m-resolution Shuttle Radar Topography Mission (SRTM) digital elevation model (DEM) provided by NASA [19] was used for terrain correction and elevation-related processing. Sentinel-1A precise orbit determination (POD) data provided by the European Space Agency (ESA), with an accuracy better than 5 cm [20], were used for baseline calculation and image coregistration. External atmospheric correction products such as GACOS are used in some SBAS-InSAR studies [21]; however, no GACOS product was applied in this study. Atmospheric signals were treated only through the internal spatiotemporal filtering implemented in the SBAS workflow, as detailed in Section 2.3.2.

### 2.3. InSAR Processing Methodology

#### 2.3.1. D-InSAR for the Initial-Mining Stage

Considering the limited number of images and the relatively rapid deformation during the 2301-1 initial-mining stage, differential interferometric SAR (D-InSAR) was adopted to obtain the spatial distribution characteristics of the subsidence basin in this stage [22]. The basic principle of D-InSAR is to invert surface displacement by extracting the deformation component from the interferometric phase, using two SAR images acquired over the same area at different times. The interferometric phase of the two acquisitions is taken as the combined contribution of several spatial components:

*φ* = *φ*_flat_ + *φ*_topo_ + *φ*_disp_ + *φ*_atmo_ + *φ*_noise_ (1)

where *φ*_flat_, *φ*_topo_, *φ*_disp_, *φ*_atmo_, and *φ*_noise_ denote the flat-earth phase, topographic phase, deformation phase, atmospheric delay phase, and noise phase, respectively. The flat-earth phase is removed using precise orbit data, and the topographic phase is removed using an external DEM. Residual low-frequency background is treated empirically by the stable-area trend-surface procedure described below; no independent GACOS atmospheric correction was applied. The remaining deformation phase is used to solve for the line-of-sight (LOS) deformation *d*_los_:

(2) $$\varphi_{\mathrm{disp}}=\varphi -\varphi_{\mathrm{flat}}-\varphi_{\mathrm{topo}}=-\frac{4\pi }{\lambda }d_{\mathrm{los}}$$

where *λ* is the radar wavelength. In general, an InSAR measurement is the projection of the three-dimensional displacement vector onto the radar line of sight: *d*_LOS_ = *d*_u_ cos*θ*_inc_ − *d*_e_ sin*θ*_inc_ cos*φ* + *d*_n_ sin*θ*_inc_ sin*φ*, where *d*_u_, *d*_e_, and *d*_n_ are the vertical, east, and north components and *φ* is the satellite heading convention [23]. The commonly used conversion *d*_v_ = *d*_LOS_/cos*θ*_inc_ is therefore valid only when the horizontal contribution is negligible. The center-scene incidence angles were 39.28° for 2301-1 and 38.56° for 2301-2. Because only one ascending geometry was available, a unique three-component solution could not be obtained; the direct projection was retained, and its uncertainty was quantified using the available principal-profile horizontal observations as described below.

(3) $$d_{v}\approx \frac{d_{\mathrm{los}}}{\cos\theta_{\mathrm{inc}}}$$

The coal seam is nearly horizontal, but this does not imply negligible horizontal surface motion. We therefore use the LOS-to-vertical projection as an approximation. The mine reports provide profile-direction horizontal movement, which was placed on the same representative intervals and projected onto the radar horizontal look direction (azimuths 76.995° and 76.890°). Multiplication by tanθinc gives the contribution of that reported component to the vertical-projection bias. The statistics in Section 4.3 use points with |*W*_PIM_| < *T*_max_. They are component-wise sensitivity estimates, not a verified three-component displacement correction.

The surveyed profile-axis azimuths were 0.106° and 84.878° for the 2301-1 strike and dip profiles, and 0.194° and 84.465° for the corresponding 2301-2 profiles (Supplementary Table S4). These axial bearings were obtained from the dominant direction of the survey east–north coordinates, clockwise from survey-grid north and modulo 180°. The source reports specify the Beijing 1954 coordinate system, with Y east and X north. Assuming approximate alignment of survey-grid north with the radar azimuth reference, the absolute projections were calculated as

|*u*_look_| = |*u*_p_cos(*ψ*_look_ − *α*_p_)|, *B*_v_ = |*u*_look_|tan*θ*_inc_ (4)

where *u*_p_ is the reported profile-direction horizontal movement, *α*_p_ is the profile-axis azimuth, *ψ*_look_ is the radar horizontal look azimuth, *θ*_inc_ is the incidence angle, and *B*_v_ is the magnitude of the vertical-projection bias contributed by that component. Under the Gauss–Krüger projection consistent with the archived coordinate pairs (Krassowsky 1940 ellipsoid; central meridian 112.5° E), absolute grid convergence was below 0.005° at the 65 archived points (Supplementary Information S4). This supports approximate alignment under that projection, but the original survey-frame orientation and any additional local rotation have not been independently verified. Reversing the movement sign or the profile axis by 180° leaves these magnitudes unchanged; an arbitrary north-reference rotation does not. The reports define south-to-north movement as positive on the strike profiles, but the results here remain absolute single-component sensitivity estimates, not signed corrections or complete three-dimensional error bounds.

The cumulative D-InSAR result contained a slowly varying long-wavelength background superposed on the mining deformation. Because no independent atmospheric-delay product or ramp-specific control data were available, its physical origin could not be uniquely separated among residual atmospheric, orbital, DEM/geocoding, terrain-correlated, and other low-frequency effects. We therefore treated it as an empirical background under an explicit stable-ground zero-deformation assumption. After phase unwrapping and LOS-to-vertical projection, a second-order trend-surface correction was applied to the archived uncorrected cumulative raster. A regional trend surface was fitted with the following quadratic polynomial:

*T*(*x*,*y*) = *a*_0_ + *a*_1_*x* + *a*_2_*y* + *a*_3_*x*^2^ + *a*_4_*xy* + *a*_5_*y*^2^ (5)

where *T*(*x*, *y*) is the trend-surface value at pixel (*x*, *y*), and *a*_0_–*a*_5_ are least-squares coefficients. Before rejection, 66,113 candidate stable pixels had finite values in all 11 pairs, mean coherence ≥ 0.50, and locations outside the working face/goaf footprint plus a 300 m buffer. Iterative 2*σ* rejection retained 52,303 pixels for the quadratic fit, which was subtracted from the uncorrected raster. Coordinates were expressed in kilometers east and north of the mapped working face center. A first-order fit was also computed as a sensitivity benchmark; the coefficients, rejection history, and diagnostics are supplied in Supplementary Data S1, and the profile-level order sensitivity is reported in Section 4.3.

#### 2.3.2. SBAS-InSAR for the Repeated-Mining Stage

Considering the abundant imagery and continuous deformation accumulation during the 2301-2 repeated-mining stage, SBAS-InSAR processing was performed in SARscape 5.6.2 using 38 ascending Sentinel-1A images acquired from 30 June 2018 to 17 September 2019. The connection graph used temporal baselines of 0–36 days and a maximum normal baseline equal to 30% of the critical baseline. It contained 108 interferograms, with a maximum selected absolute normal baseline of 209.97 m. All 38 acquisitions formed one connected network; each acquisition had 3–6 links (mean 5.68, with a requested maximum of 8), and 13 February 2019 was selected as the super master. Precise orbit files and the 30 m SRTM DEM were used during coregistration. Multilooking factors were 4 in range and 1 in azimuth. These settings follow established small-baseline time-series practice [5,24].

Spectral-shift filtering was enabled during interferogram generation. The interferometric phase was then filtered using the Goldstein method with a 48-pixel window, 40% overlap, and a 5 by 5-pixel coherence-estimation window. Phase unwrapping used the minimum-cost-flow Delaunay method with a coherence threshold of 0.30. The weighted singular-value-decomposition inversion used the same coherence threshold and required at least 65% valid interferograms; the second inversion also required at least three interferograms per acquisition. Residual atmospheric components were estimated internally using a 1600 m spatial low-pass filter and a 365-day temporal high-pass filter. Automatic refinement masks were used for residual-phase referencing rather than a manually specified reference point or polygon. The cumulative displacement series was temporally referenced to the first acquisition on 30 June 2018. Final products were geocoded to WGS 84 at 15 m by 15 m pixel spacing; a temporal-coherence threshold of 0.10 was applied during output generation.

Let the *N* + 1 SAR images covering the study area be ordered by acquisition time as *t*_0_, *t*_1_, …, *t_N_*. Based on the baseline thresholds, they are combined pairwise to generate *M* small-baseline differential interferograms, the number of which satisfies:

(6) $$\frac{N+1}{2}\leq M\leq \frac{N(N+1)}{2}$$

For the *j*-th interferogram (*j* = 1, 2, …, *M*) generated from the images at times *t*_A_ and *t*_B_, and after removing the topographic phase with a DEM and attenuating the atmospheric and noise components by filtering, its differential phase mainly reflects the difference in cumulative LOS deformation between the two times. Displacement is approximated as piecewise linear between acquisitions, with an average velocity assigned to each interval. The differential phase is then expressed through the time integral of these interval velocities:

(7) $$\varphi_{j}\approx \frac{4\pi }{\lambda }(d_{A}-d_{B})=\frac{4\pi }{\lambda }\sum_{k=t_{A}+1}^{t_{B}}\left(t_{k}-t_{k-1}\right)v_{k}$$

where *d*_A_ and *d*_B_ denote the line-of-sight (LOS) cumulative deformation at times *t*_A_ and *t*_B_ relative to the reference time, respectively, and *v_k_* is the average deformation rate during the *k*-th time interval. The *M* interferometric-pair equations are combined into the matrix form ***Bv*** = ***φ***, where ***B*** is the *M* × *N* coefficient matrix. Although disconnected small-baseline subsets can make ***B*** rank-deficient in general, all 38 acquisitions in the present study formed one connected network. Weighted singular value decomposition (SVD) was nevertheless used to compute its generalized inverse and obtain the interval deformation rates in the least-norm sense, after which the cumulative deformation at each epoch was obtained by temporal integration. On this basis, SBAS-InSAR time-series processing was applied to the Sentinel-1A images of the repeated-mining stage to retrieve the cumulative evolution of surface subsidence.

Similarly to the initial-mining stage, SBAS-InSAR can characterize the temporal expansion trend at the basin margin; however, decorrelation holes still appear in the central high-gradient area due to coherence loss, making it difficult to recover the complete central subsidence magnitude. PIM was therefore introduced to estimate deformation in the basin center [25].

### 2.4. Probability Integral Method

The probability integral method (PIM) represents the overburden as a random medium at the macroscopic scale [26,27]. A Gaussian influence kernel describes the contribution of an extracted element, and its integration over the finite panel gives the surface subsidence field [28]. The finite-panel profile is an integral of the Gaussian kernel and need not itself be Gaussian or form an elliptical plan-view basin.

Let *x* and *y* denote local strike and dip coordinates in the numerical data. Equation (8) gives a general separable PIM formulation; the near-horizontal, single-radius reduction used here is specified below:

(8) $$\left\{\begin{array}{l} W_{\max}=m\cdot q\cdot \cos\alpha \\ l=L_{0}-S_{1}-S_{2}\\ L=(L_{1}-S_{3}-S_{4})\cdot \frac{\sin(\theta +\alpha )}{\sin\theta }\\ W^{0}(x)=\frac{W_{\max}}{2}\cdot \left[\mathrm{erf}\sqrt{\pi }\frac{x\cdot \tan\beta }{H_{0}}-\mathrm{erf}\sqrt{\pi }\frac{(x-l)\cdot \tan\beta }{H_{0}}\right]\\ W^{0}(y)=\frac{W_{\max}}{2}\cdot \left[\mathrm{erf}\sqrt{\pi }\frac{y\cdot \tan\beta }{H_{1}}-\mathrm{erf}\sqrt{\pi }\frac{(y-L)\cdot \tan\beta }{H_{2}}\right]\\ W(x,y)=\frac{1}{W_{\max}}\cdot W^{0}(x)\cdot W^{0}(y) \end{array}\right.$$

For numerical reporting, *W*_max_ = 1000 mq cosα is the theoretical amplitude in millimeters when the mining thickness m is in meters; *q* = *W*_max_/(1000 m cosα). It is not calculated from the observed peak of a finite panel. *L* and *D* are the extracted strike length and panel width, *α* is seam dip, and *r* = *H*/tan*β* is the influence radius. A representative depth *H* = 390 m is used to report tan*β*; the inversion estimates *r* directly. *S*_1_ and *S*_2_ refer to the *y* = 0 and *y* = *D* sides, and *S*_3_ and *S*_4_ to *x* = 0 and *x* = *L*. To obtain the implemented kernel from the two-profile product in Equation (8), the first profile maps to the dip direction and the second to strike, each measured from its first effective boundary. Effective dimensions are the recorded panel dimensions less the opposing offsets. The reduction uses a common influence radius and omits the propagation-angle length projection; neither a propagation angle nor direction-specific depths are estimated. The positive-meter profile product is converted to downward-negative millimeters. Table 4 summarizes the numerical-model symbols.

Supplementary Table S2 defines the additional symbols in the general formulation of Equation (8) and distinguishes its profile coordinates and effective lengths from the numerical-model notation in Table 4.

Equation (9) groups the geometry and empirical quantities of the general formulation. The reduced numerical model instead fits the subsidence factor, a common influence radius, and the constrained offsets. Model A uses one common offset. Model B has two dip-side offsets and imposes *S*_3_ = *S*_4_ = (*S*_1_ + *S*_2_)/2; the four offsets are therefore not all fitted independently.

(9) $$\left\{\begin{array}{l} \mathrm{GP}=[m,\alpha ,H_{0},H_{1},H_{2},L_{0},L_{1}]\\ P=[q,\tan\mathrm{tan\beta},\theta ,S] \end{array}\right.$$

The static field is evaluated from the known geometry GP and the chosen low-dimensional parameter set P. In this numerical evaluation, these sets contain the recorded panel dimensions, mining thickness and seam dip, and the reduced fitted variables described above:

*W*(*x*,*y*) = *f*(**GP**,**P**) (10)

A campaign development factor separates spatial shape from temporal realization: *Ŵ_t_*(*x*,*y*) = *k_t_ W*_static_(*x*,*y*;Lt,*D*,P), where Lt is recorded mining advance and downward displacement is negative. Conditional on the spatial parameters, weighted isotonic least squares determines 0 ≤ *k*_1_ ≤ … ≤ *k_T_* = 1 from the field observations. The final campaigns, 26 July 2016 and 18 September 2019, define the normalization *k_T_* = 1; they are not independently demonstrated to represent ultimate subsidence. Replacing k by ak and *q* by *q*/*a* leaves every prediction unchanged. Thus, *q* is a reconstruction parameter conditional on this normalization rather than a uniquely identified ultimate subsidence coefficient.

The spatial objective contains equal-weight field residuals from three late campaigns: 28 May, 28 June, and 26 July 2016 for 2301-1; and 29 January, 21 February, and 18 September 2019 for 2301-2. The full 21/12-campaign sequences determine the monotone factors but do not all enter the spatial objective. Campaigns with *L* ≤ *S*_3_ + *S*_4_ have crossed effective strike boundaries and are excluded from factor estimation for that trial. The broad numerical bounds are 0.01 ≤ *q* ≤ 2, 48.75 ≤ *r* ≤ 780 m, and 0 ≤ *S*_1_,*S*_2_ ≤ 0.49*D*; *q* is not fixed. Models A and B were compared using identical fivefold spatial blocks at the representative epochs, preferring the lower-dimensional model if their RMSEs differed by less than 2%. This is a practical selection rule, not a significance test. The selected fits used 48 deterministic full-data starts and 24 starts per fold. Joint field-plus-InSAR fits and omission of the final campaign were secondary sensitivity checks. Parameter estimation was performed using the lsqnonlin solver in MATLAB R2022b.

Parameter uncertainty was explored using 500 spatial-block bootstrap resamples per stage. Each profile was partitioned into the same five contiguous blocks used in the spatial assessment; five blocks were sampled with replacement within each profile. All campaigns of a monument received the same block multiplicity, and the spatial parameters and campaign factors were refitted. The selected model, recorded geometry, parameter bounds and terminal-factor normalization were held fixed. The 2.5th and 97.5th percentiles summarize conditional sampling variability; they do not include model-selection, geometry or normalization uncertainty. Bound-limited resamples were retained. Supplementary Table S3 reports the intervals and boundary-hit frequencies, with the samples and fitting script in Supplementary Data S1.

The secondary inversion adds 15 perimeter samples per stage from the processed InSAR products: the trend-corrected *D*-InSAR grid on 23 May 2016 and the non-IDW SBAS product on 25 February 2019. Candidates had coherence ≥ 0.50, boundary distance 30–250 m outside the panel, and downward magnitude between 10 mm and *T*_min_ (95/103 mm). *D*-InSAR samples were finite in all 11 coherence rasters. SBAS additionally required image percentage ≥ 90%, valid-interferogram percentage ≥ 65%, and velocity precision ≤ 8 mm year^−1^. A deterministic perimeter-coverage score selected 15 points with at least 75 m separation. These samples were not included in the primary field-only fit or its spatial-block predictions. Their small fixed number permits a controlled comparison with the 195/186 late-campaign field residuals.

The representative stage-1 prediction is *k*_28May_ *W*_static_(*L* = 996.4 m) − 0.8*k*_1_3July *W*_static_(*L* = 392 m) − 0.2*k*_28July_ *W*_static_(*L* = 428 m). The start prediction is therefore interpolated between complete modeled displacement states, exactly as for the observed start state; evaluating the nonlinear model once at an interpolated advance is not equivalent. The stage-2 prediction is *k*_21February_ *W*_static_(*L* = 996.9 m). Local mine coordinates were registered to the mapped InSAR coordinates using the 65 paired stage-1 coordinates, with no displacement values in the registration; its vector RMS residual was 0.27 m. The same registration was used for both stages. This is a local spatial alignment, not a certified geodetic datum transformation.

### 2.5. Dynamic Weighting Fusion Framework

**Figure 4:**
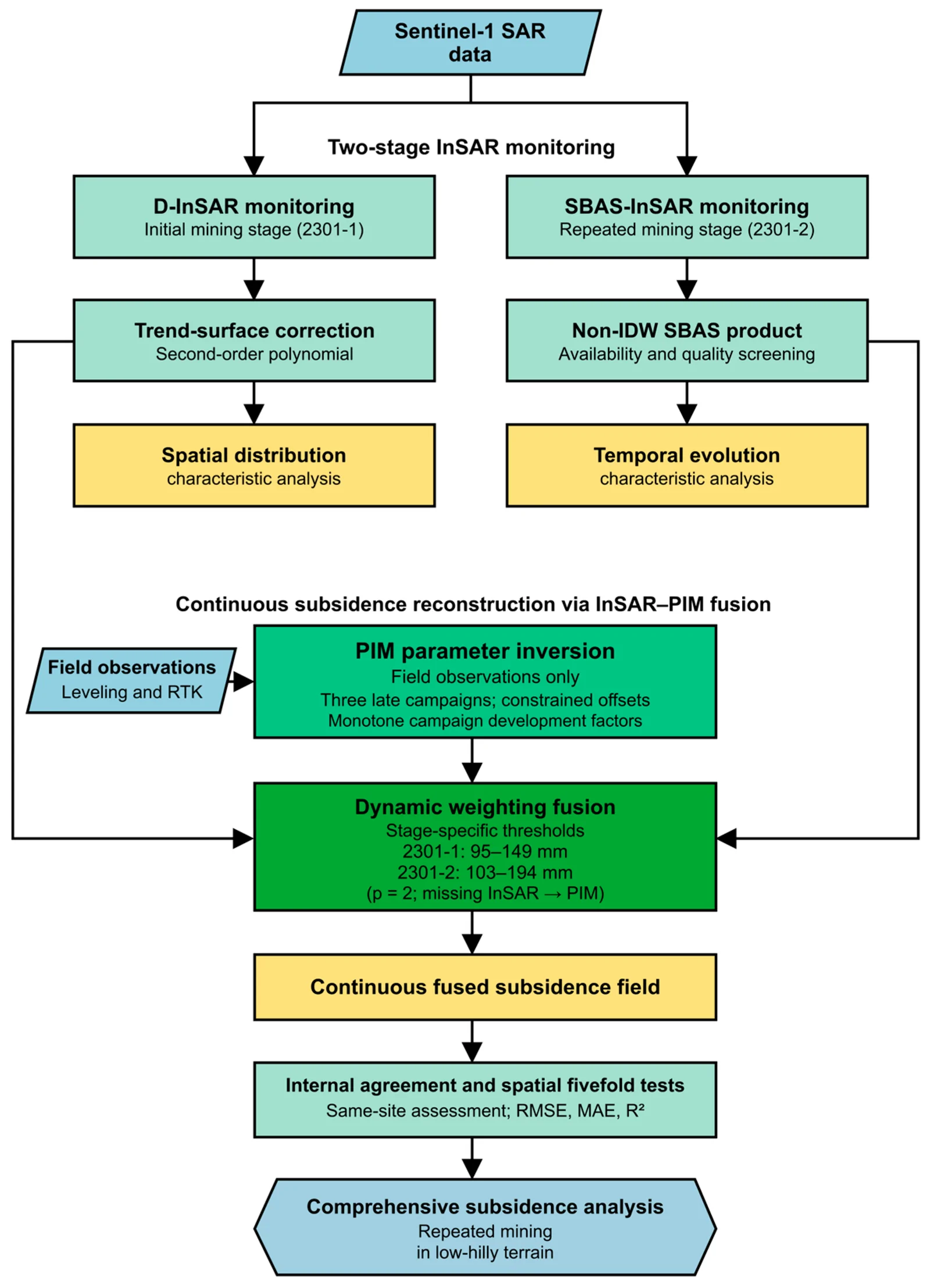
Technical workflow of the proposed two-stage InSAR–PIM fusion framework for continuous subsidence reconstruction.

The proposed method addresses large surface subsidence gradients, deformation asymmetry, and the susceptibility of InSAR to decorrelation under repeated-mining conditions in low-hilly terrain. It builds on previous studies about InSAR monitoring, PIM inversion, and dynamic weighting [29,30,31] and uses a three-part workflow comprising “stage-wise monitoring–PIM parameter inversion–dynamic weighting fusion” (Figure 4).

Stage-specific InSAR processing supplies the basin-margin observations and the empirical thresholds. Field-only PIM inversion supplies the finite-panel displacement field, including the central decorrelation zone. Dynamic weighting then blends the two sources. The secondary joint inversion quantifies the incremental contribution of InSAR perimeter samples to parameter estimation; the primary reconstruction uses the field-only estimates.

The dynamic weighting scheme uses deformation magnitude to avoid the step effect associated with hard-threshold stitching at the fusion boundary. The magnitude of the PIM-predicted subsidence controls the relative weights assigned to InSAR and PIM. The symmetric power-ratio weighting function is expressed as:

(11) $$P_{\mathrm{InSAR}}=\left\{\begin{array}{cc} 1 & A\leq T_{\min}\\ \frac{{\left(T_{\max}-A\right)}^{p}}{{\left(T_{\max}-A\right)}^{p}+{\left(A-T_{\min}\right)}^{p}} & T_{\min}\leq A\leq T_{\max}\\ 0 & A\geq T_{\max} \end{array}\right.$$

Subsequently, the final fused subsidence value *W*_fusion_ is obtained using the data fusion reconstruction formula, Equation (12):

*W*_fusion_ = *P*_InSAR_ ⋅ *W*_InSAR_ + (1 − *P*_InSAR_) ⋅ *W*_PIM_ (12)

where *W*_PIM_ is the signed PIM-predicted subsidence and *A* = |*W*_PIM_| is the nonnegative weight-controlling magnitude; *T*_min_ and *T*_max_ delimit the transition, *p* controls its shape, and *W*_InSAR_ is the signed InSAR observation. Equation (12) combines the signed displacements; only the weight calculation uses *A*. For a product-valid cell, *P*_InSAR_ = 1 when *A* ≤ *T*_min_, *P*_InSAR_ = 0 when *A* ≥ *T*_max_, and Equation (11) gives a continuous decrease between the thresholds. *A* validity override is applied before Equation (12). If no InSAR source lies within 24 m of the 15 m output cell, or if the associated source fails the final-product availability criteria, the effective InSAR weight is set to zero and PIM supplies that cell regardless of *A*. Availability required a finite corrected value and all 11 pairs for D-InSAR; for SBAS it required a finite displacement, temporal coherence ≥ 0.10, image percentage ≥ 90%, valid-interferogram percentage ≥ 65%, and velocity precision ≤ 8 mm year^−1^. The general SBAS output cutoff remained unchanged; the stricter threshold of 0.50 was used only to select the 15 auxiliary perimeter samples by the preceding deterministic procedure.

The deformation thresholds define an operational transition interval and are not universal sensor limits. *T*_min_ marks the onset of model blending and *T*_max_ marks the adopted empirical retrieval limit for the corresponding processed product. Because the two stages use different InSAR strategies and error structures, their thresholds were calibrated separately using the operational procedures reported in Section 3.1.3 and Table 5. Section 3.5 evaluates these case-specific heuristics using nine ±10% threshold configurations.

The power parameter *p* controls the sharpness of the symmetric transition around *T*_mid_ = (*T*_min_ + *T*_max_)/2, where the two sources receive equal weight. For *p* = 1, the weight decreases linearly; for *p* > 1, its derivative with respect to *A* tends to zero at both *T*_min_ and *T*_max_, matching the constant outer branches. Thus, the weight is continuously differentiable at both thresholds. This removes threshold-induced value and first-derivative jumps when the source fields are differentiable but does not eliminate their local gradient peaks or discontinuities at availability-mask boundaries. We retained *p* = 2 as a moderate nonlinear transition. Section 3.5 evaluates *p* = 1, 1.5, 2, and 3; larger *p* concentrates the change near the midpoint.

For direct benchmarking, hard-threshold stitching switched from InSAR to PIM at *T*_mid_ = (*T*_min_ + *T*_max_)/2, while the fixed-weight benchmark used equal weights (0.5/0.5) throughout the transition interval and the same pure-source rules outside it. Continuity was evaluated after piecewise-linear interpolation of each original profile to a 0.25 m grid, which avoids making the diagnostic depend on how many field-observation points happened to fall inside the narrow transition. We report the theoretical splice-value jump at threshold crossings and the maximum absolute local gradient around those crossings, together with pointwise RMSE on the original field-observation locations. The maximum absolute local gradient was calculated from adjacent samples within ±1 m of the *T*_min_, *T*_mid_, and *T*_max_ crossings. This assessment concerns the magnitude-threshold crossings, not boundaries introduced by the InSAR availability mask.

Full-data representative-profile statistics use the same 65/62 monuments as the multi-epoch fit and therefore measure internal reconstruction agreement. Each principal profile was divided into five contiguous distance blocks for spatial testing. In each fold, all records for the withheld monument identifiers were removed from every campaign, after which all spatial parameters and campaign factors were refitted on the remaining field data. No InSAR perimeter observations entered these primary fits. The withheld monuments were then predicted at the representative interval. This prevents cross-epoch leakage. The same folds also informed selection between models A and B, introducing possible selection bias. The reported values therefore describe same-site spatial cross-validation after model selection, not independent external performance.

Neighboring monuments along a profile are spatially dependent. Consequently, the coefficients of determination summarize agreement over these sampled profiles and should not be interpreted as evidence from independent observations or as an unbiased measure of prediction at new sites. Contiguous-block withholding reduces local information sharing but does not remove all spatial dependence.

For 2301-2, *W*_PIM_ represents displacement accumulated after the 6 July 2018 field baseline, whereas SBAS begins on 30 June. The extra six SBAS days precede lower-slice extraction but may contain residual compaction or other background motion. After 6 July, both fields may include lower-slice-induced deformation and residual compaction or renewed deformation of the upper and neighboring goafs, including 2303. The single PIM field and its magnitude-based fusion weight do not separate these mechanisms.

## 3. Results

### 3.1. InSAR Monitoring Results

#### 3.1.1. D-InSAR Results for the Initial-Mining Stage

For the 2301-1 initial-mining stage, D-InSAR differential processing was reconstructed from the archived same-track ascending Sentinel-1A project. Adjacent retained acquisitions were used wherever a processed interferogram was available. With 16 July 2015 as the zero-deformation reference, the final cumulative chain used 12 scenes and 11 interferometric pairs: nine 24-day pairs and two 48-day pairs (31 December 2015–17 February 2016 and 5 April–23 May 2016). The first 48-day link spans a gap for which no intervening scene was present in the source set. For the second, the archive contains a 29 April 2016 SLC but no retained interferogram involving it; consequently, the 5 April–23 May link is the next available connection in the archived processing chain. This later 48-day pair had the lowest mean coherence (0.379), indicating increased temporal decorrelation, whereas the earlier 48-day pair retained a mean coherence of 0.611. Longer temporal baselines contributed to the loss of coherence, although other factors were also involved. The temporal and perpendicular baseline parameters are listed in Table 2.

**Figure 5:**
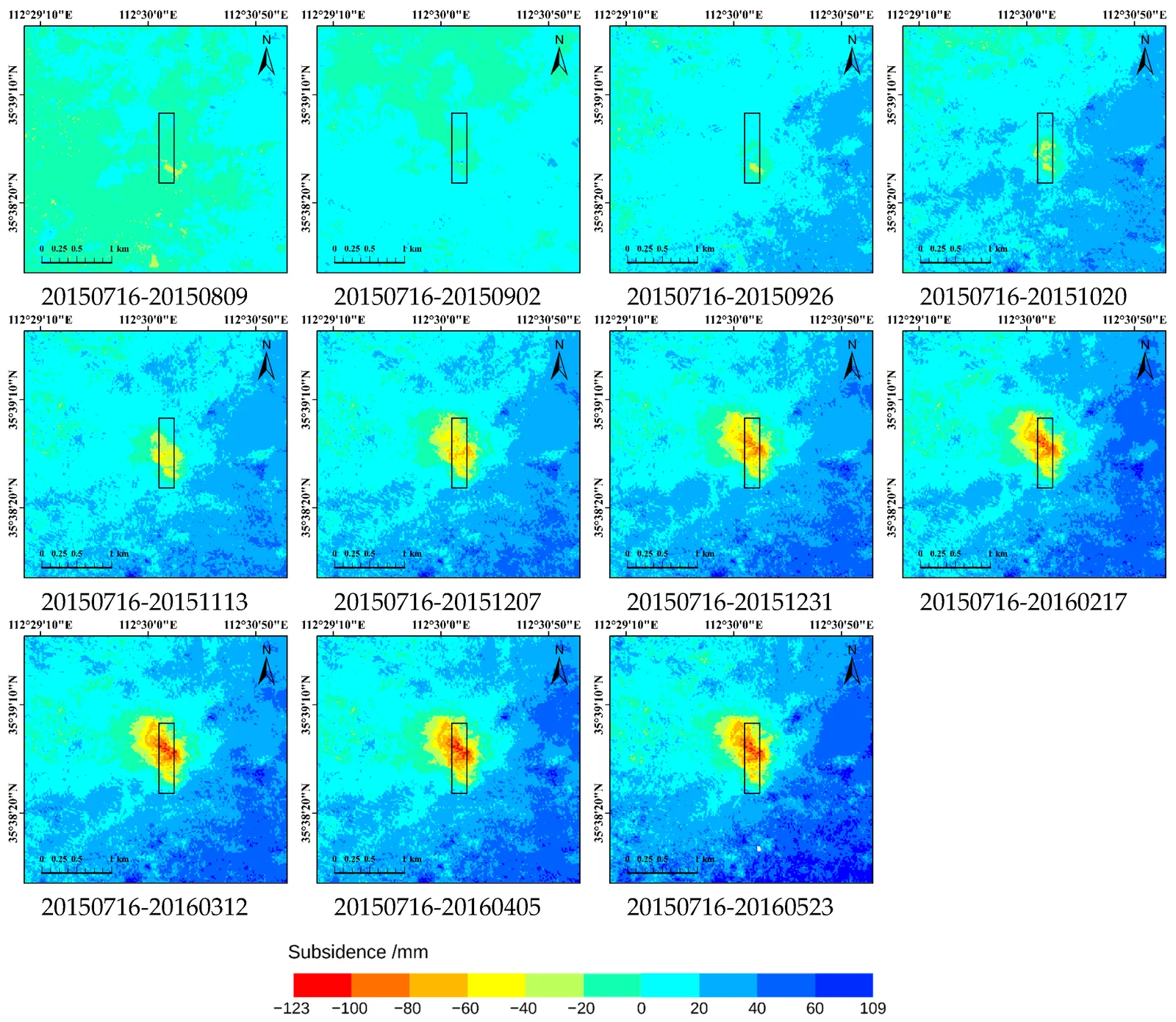
Timeseries DInSAR-derived cumulative subsidence maps for the initial-mining stage (2301-1), referenced to 16 July 2015.

As shown in Figure 5, subsidence was small and spatially limited during the early monitoring period. As mining advanced, cumulative subsidence increased and the basin expanded, indicating continued ground movement during the study period. Coherence remained high at basin margins, where subsidence gradients were gentle and deformation was continuous. In the high-gradient center, however, the signal weakened, and later acquisitions contained contiguous gaps and anomalous values. This degradation was likely caused by the large deformation gradients during active mining, compounded by vegetation cover and terrain relief, which reduced the coherence of the Sentinel-1A C-band observations.

**Figure 6:**
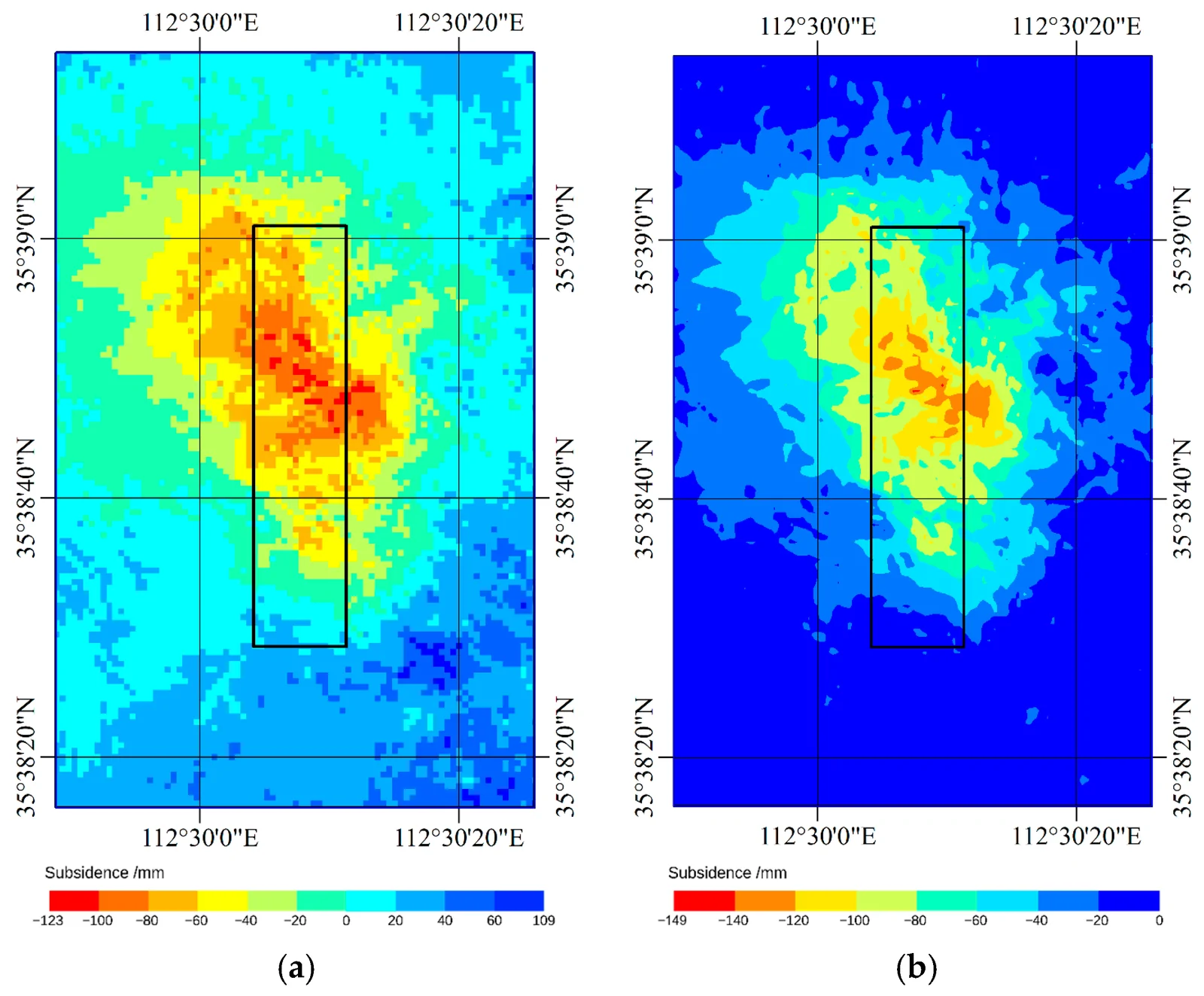
D-InSAR-derived cumulative subsidence maps for the initial-mining stage (2301-1). (**a**) Original cumulative subsidence. (**b**) Reprocessed empirical trend-surface-corrected cumulative subsidence.

Figure 6 shows the cumulative D-InSAR subsidence results for the 2301-1 working face (16 July 2015–23 May 2016). Figure 6a presents the archived uncorrected cumulative raster, in which a subsidence funnel formed above the working face but a long-wavelength background displaced stable-ground values from zero. Among the 66,113 candidate stable pixels, the mean/RMSE relative to the zero-deformation assumption changed from +34.03/39.70 mm before correction to −0.16/9.36 mm after the reproduced quadratic correction. This demonstrates the intended stable-area centering but does not identify the physical source of the removed component. The reprocessed source-grid minimum was −149.29 mm (Figure 6b), compared with −123.28 mm before correction; the rounded magnitude of 149 mm was adopted as the stage-specific D-InSAR empirical effective retrieval limit (Section 3.1.3). C-band decorrelation still prevented reliable recovery of the much larger central subsidence.

#### 3.1.2. SBAS-InSAR Results for the Repeated-Mining Stage

**Figure 7:**
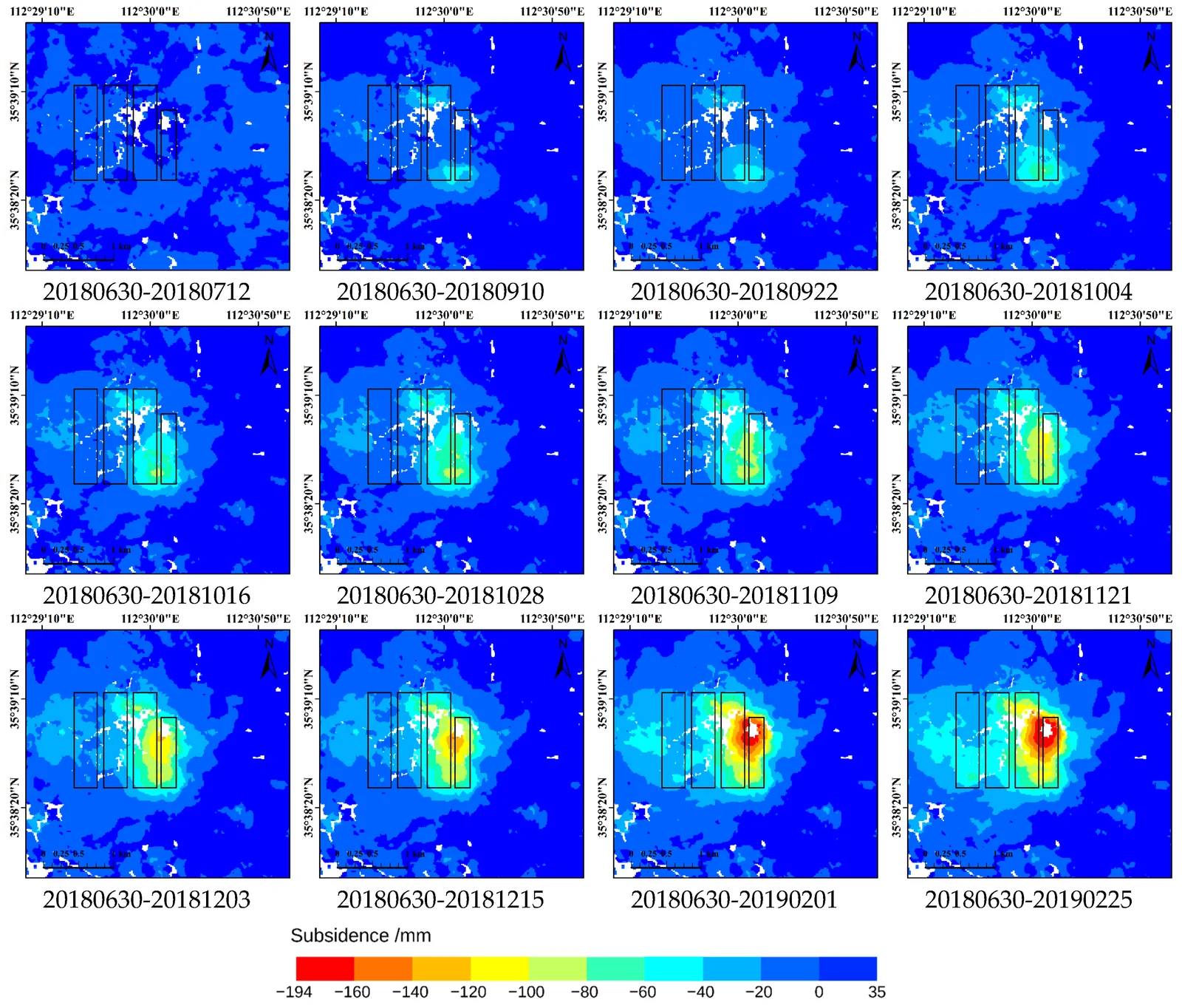
Time-series SBAS-InSAR-derived cumulative subsidence maps for the repeated-mining stage (2301-2).

To obtain continuous time-series deformation information for the repeated-mining stage, SBAS-InSAR processing was applied to the multi-temporal Sentinel-1A images according to the method described in Section 2.3.2. For the 2301-2 repeated-mining stage, 38 Sentinel-1A images acquired from June 2018 to September 2019 were selected to construct a small-baseline interferometric network, and time-series inversion was performed using 30 June 2018 as the initial reference to obtain the temporal evolution of cumulative subsidence in the study area. Because mining of the 2301-2 working face ended in January 2019 and the main subsidence process had largely been completed by February 2019, the cumulative subsidence result on 25 February 2019 was selected as the representative subsidence field for the repeated-mining stage for subsequent analysis (Figure 7).

As shown by the temporal evolution in Figure 7, subsidence was initially limited in magnitude and spatial extent. As the working face advanced, the basin expanded and migrated along strike, and cumulative subsidence increased. By February 2019, large displacements were concentrated in the basin center. The repeated-mining representative field has a larger observed subsidence magnitude than the initial-mining interval field; the differing reference periods and possible residual compaction prevent this comparison from isolating a net repeated-mining amplification effect.

The time series retains coherent observations near the margins but contains gaps in the high-gradient center. The 21 February 2019 field survey has a reconstructed peak magnitude of 1743 mm, compared with the approximately 194 mm empirical limit of the 25 February SBAS field. The four-day offset is retained; the field endpoint was not interpolated to the SAR date.

**Figure 8:**
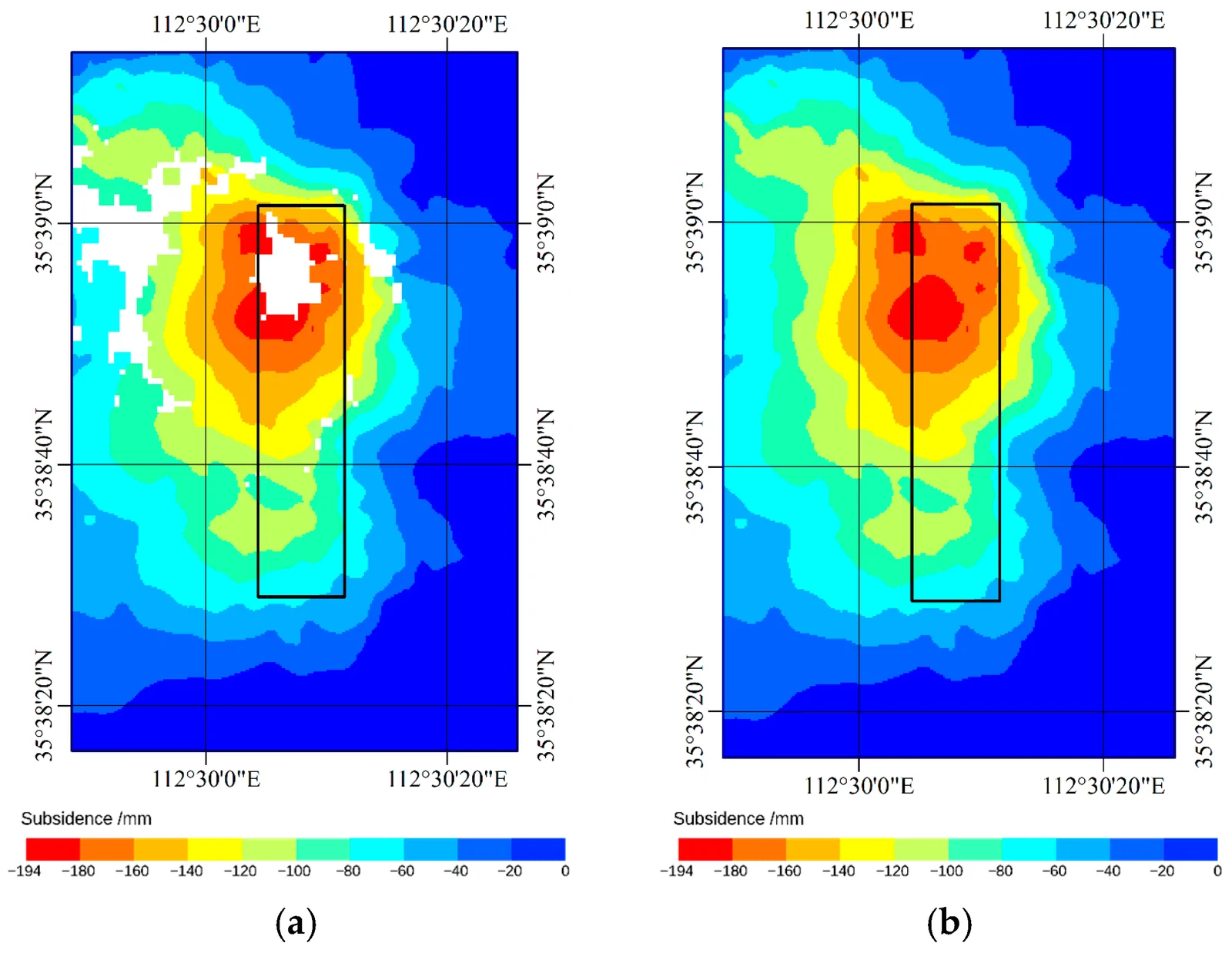
SBAS-InSAR-derived cumulative subsidence maps for the repeated-mining stage (2301-2). (**a**) Before IDW interpolation. (**b**) After IDW interpolation.

For visual comparison only, inverse distance weighting (IDW) was applied to illustrate how small local SBAS gaps could be filled (Figure 8b). The quantitative margin constraints, profile comparisons, and final fusion grids did not use this IDW field. They used the non-interpolated final SBAS product, associated with the 15 m fusion grid by nearest-source mapping within 24 m; cells without an available source were handled by the zero-weight override in Section 2.5. IDW cannot reconstruct the extensive high-gradient center, which remains PIM-controlled.

#### 3.1.3. InSAR Retrieval Capability and Empirical Retrieval Limits

The detection capability of both D-InSAR and SBAS-InSAR is jointly constrained by the radar wavelength, spatial sampling scale, and phase-unwrapping capability. When the deformation difference between adjacent pixels exceeds the detectable phase-gradient limit, phase ambiguity and unwrapping errors may occur in the interferometric phase [32]. This theoretical relationship can be expressed as follows:

(13) $$d_{\max\;}=\frac{\lambda }{2\mu }$$

where *d*_max_ denotes the maximum deformation gradient detectable between adjacent pixels, *λ* is the radar wavelength, and *μ* is the pixel size of a single SAR image. This equation indicates that large-gradient deformation leads to phase ambiguity and unwrapping instability, and that the actual detectable threshold still needs to be determined in combination with the coherence of the study area.

In addition, high-intensity mining activities can modify the geometric characteristics of surface scatterers, resulting in coherence degradation. According to the coherence correction model proposed by Baran et al. [33]:

*D*_max_ = *d*_max_ + 0.002 (*γ* − 1) (14)

where *γ* is the interferometric coherence coefficient (0 ≤ *γ* ≤ 1), and *D*_max_ is the detectable deformation-gradient limit after the coherence term in the Baran et al. model [33]. This relation motivates coherence-dependent gradient attenuation for a single interferogram. It is not treated here as proof that cumulative limits increase linearly with stack length or that selection of a high-coherence subset is unbiased.

*W*_lim_ = *μ* ⋅ *D*_max_ ⋅ *N* (15)

where *N* denotes the number of interferograms used in the operational cumulative proxy of Equation (15). For sequential D-InSAR, this proxy was used only to define an operational transition onset; it is an empirical calibration heuristic rather than a universal physical error-propagation law. It was not applied to the redundant SBAS network.

According to Equation (13), C-band wavelength and the adopted sampling imply a small adjacent-pixel deformation-gradient tolerance; large mining gradients therefore promote ambiguity and unwrapping instability. We use the term empirical effective retrieval limit (EERL) for the largest deformation magnitude retained by the processed product before valid observations became sparse, not for an intrinsic sensor specification. The trend-corrected 2301-1 D-InSAR product gave an EERL of 149 mm, and the 2301-2 SBAS product gave 194 mm. The average coherence of the 11 D-InSAR pairs was 0.543, compared with 0.62 for the SBAS network, but the difference also reflects network design, filtering, land cover, and acquisition conditions.

On this basis, *T*_max_ was set to the case-specific EERL: 149 mm for 2301-1 and 194 mm for 2301-2. For 2301-1, six of the 11 sequential pairs exceeded the chain-average coherence; their mean *γ* was 0.605. Using consistent meter units, λ = 0.0555 m and μ = 15 m give *d*_max_ = 0.00185; γ = 0.605 gives *D*_max_ = 0.00106. With N = 6, Equation (15) gives 0.0954 m (95.4 mm), rounded to *T*_min_ = 95 mm. At a fixed accumulation count, selecting above-mean-coherence pairs raises the coherence-based gradient tolerance and may yield an optimistic transition-onset estimate relative to the full-chain coherence distribution; the smaller accumulation count does not establish that this selection effect is offset. For 2301-2, Equation (15) was not used because SBAS employs a redundant network and SVD inversion. *T*_min_ was the 95th percentile of the valid non-IDW SBAS magnitudes (102.7 mm), rounded to 103 mm. Both rules are operational calibration heuristics, and their sensitivity to threshold perturbations is evaluated in Section 3.5.

The largest reconstructed field-observation magnitudes were 1175.4 mm for the 2301-1 interval and 1743 mm for 2301-2 relative to 6 July 2018. They greatly exceed the corresponding InSAR retrieval limits. The five-day stage-1 endpoint offset, six-day stage-2 start offset, four-day stage-2 endpoint offset, and interpolated stage-1 start remain temporal uncertainties.

### 3.2. Multi-Epoch PIM Parameter Inversion and Prediction Results

PIM was fitted to the field-observation sequences reconstructed from archived heights. The primary fits contain no InSAR constraints. The representative stage-1 field is an interval increment aligned at its start to *D*-InSAR; the representative stage-2 field retains the stated six-day baseline difference from SBAS. A secondary joint inversion evaluates the additional influence of the perimeter samples.

The primary spatial objectives contain 195 and 186 field residuals from the three late campaigns. Model B was selected for 2301-1: its spatial-block PIM RMSE was 61.39 mm, compared with 90.55 mm for model A. Model A was selected for 2301-2, with 173.05 mm compared with 187.41 mm for model B. All 48 full-data starts converged to the same numerical neighborhood, and no selected full-data or fold fit reached a parameter bound. The fits were numerically repeatable under the stated assumptions; physical parameter identification is discussed in Section 4.1. Table 6 reports the primary field-only estimates.

In Table 6, *S* denotes the arithmetic mean of the four boundary offsets; the calculations retain the four individual offsets. For 2301-1, the actual calculation uses *S*_1_ = 36.02 m, *S*_2_ = 73.29 m, and *S*_3_ = *S*_4_ = 54.66 m. For 2301-2, all four offsets equal 36.51 m. Table 7 compares the primary fit with a secondary fit that adds the 15 InSAR perimeter samples without changing the model structure or field-data window.

Adding the perimeter samples changed the late-window field RMSE by less than 0.01 mm in each stage. Their own RMSE decreased by 0.03 mm in 2301-1 and 0.01 mm in 2301-2. Thus, these particular perimeter observations have little incremental influence on the spatial inversion. The central magnitude is constrained primarily by the field observations.

**Figure 9:**
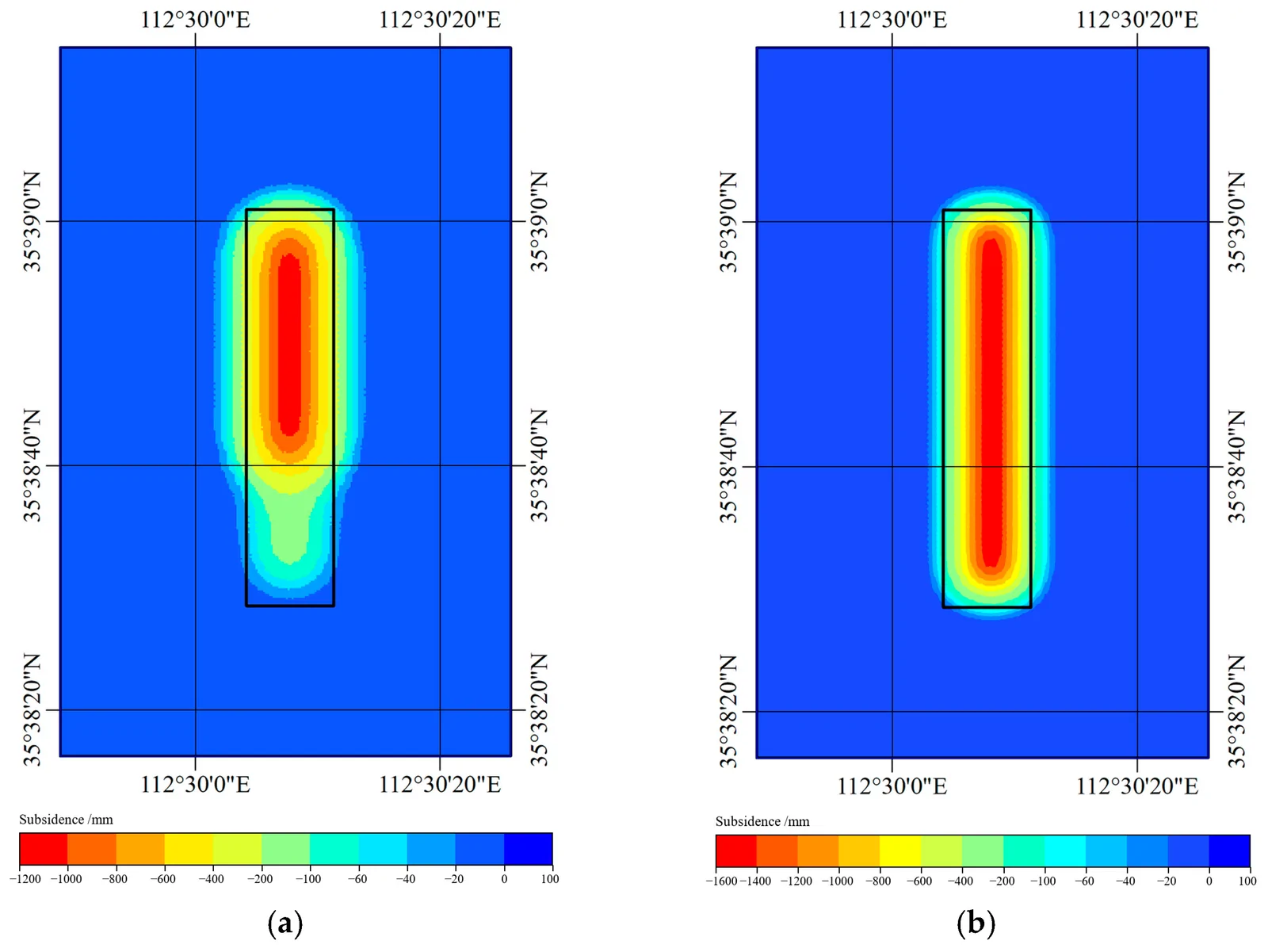
Representative PIM fields from the field-only multi-epoch fits. (**a**) 2301-1 increment from the interpolated 16 July 2015 state to 28 May 2016. (**b**) 2301-2 cumulative field on 21 February 2019 relative to 6 July 2018. Rectangles show working face footprints.

The selected parameters and campaign factors were substituted into the representative-time operators in Section 2.4. Figure 9a maps the 2301-1 end-minus-interpolated-start increment; Figure 9b maps the 2301-2 cumulative displacement relative to 6 July 2018. The complete factors and full-precision parameters are provided in Supplementary Data S1.

The stage-1 interval field combines deformation above the newly extracted segment with continued movement above previously extracted ground. Its directional asymmetry follows from subtracting the interpolated earlier panel states; a single elliptical basin is not expected. PIM and fusion were evaluated on the existing 15 m calculation grid. For display only, PIM was interpolated by IDW (power 2; 12 neighbors; spacing 4.3288 × 10−5°), and InSAR was bilinearly resampled to that display grid. The same weighting function was evaluated on that display grid, with nearest-neighbor resampling of the availability mask. No display-interpolated points were added to the accuracy assessment.

The theoretical amplitudes *W*_max_ are 2330.6 and 2113.4 mm. They differ from the finite representative-field peaks because of panel geometry, development factors, and the stage-1 interval subtraction. The calculation-grid minima in Figure 9 are −1116.3 and −1564.9 mm, respectively.

The corresponding influence radii are 125.75 and 91.93 m. At the common reference depth of 390 m these give tan*β* = 3.101 and 4.242. The depth and tan*β* are not independently identified by this inversion; the spatial kernel depends on their ratio through *r*.

InSAR supplies observed spatial variation over product-valid margins, while field-constrained PIM fills the high-gradient center. This complementary spatial coverage is the role of InSAR in the fusion, despite the limited influence of the 15 perimeter samples on PIM parameter estimation.

### 3.3. Fusion Reconstruction Results

**Figure 10:**
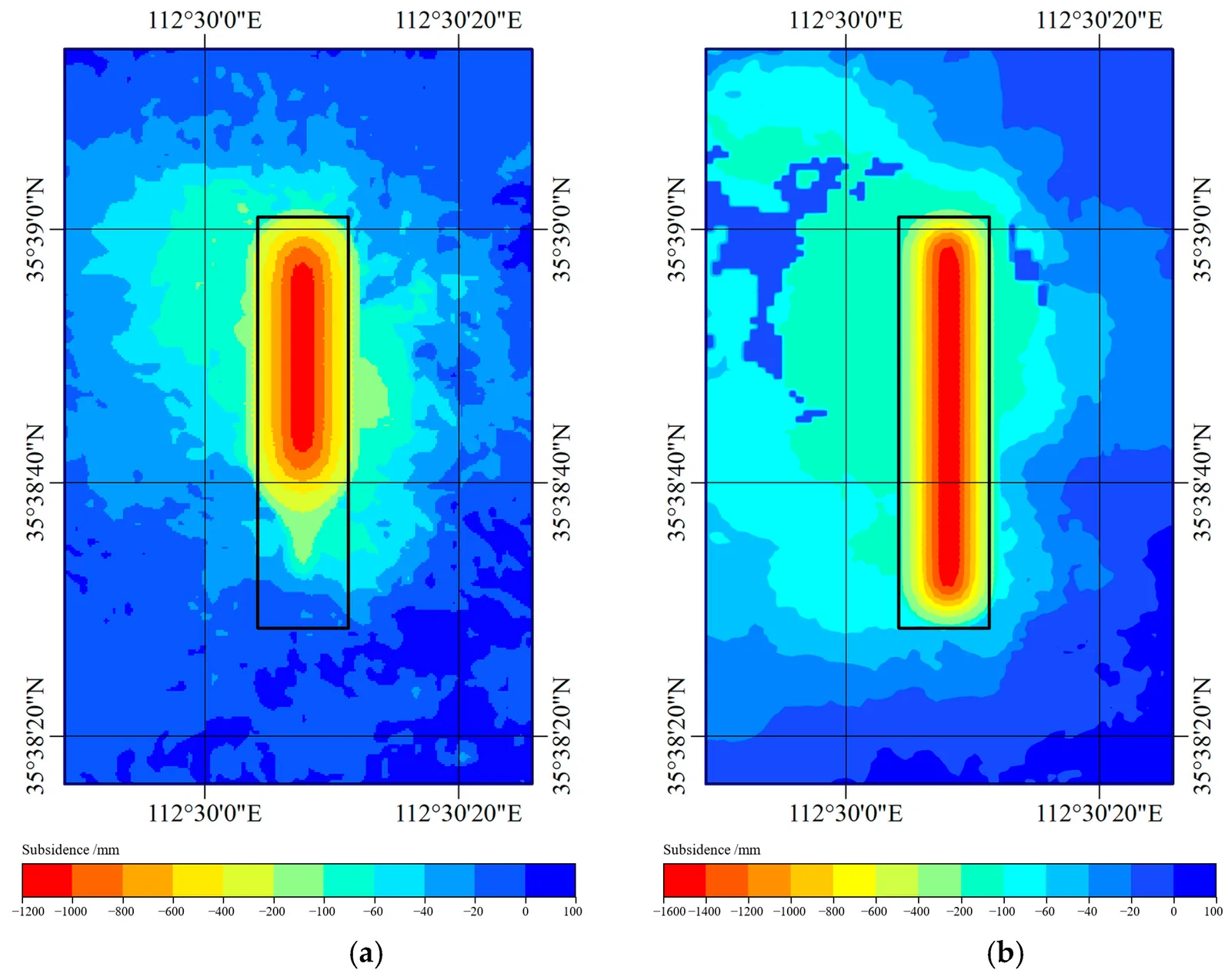
Fused fields using multi-epoch PIM predictions and InSAR availability screening. (**a**) 2301-1 interval increment; (**b**) 2301-2 cumulative field. Product-invalid cells use PIM (effective InSAR weight = 0).

Figure 10 shows the fields reconstructed by dynamic weighting. For product-valid cells, the observed InSAR value is retained in the low-magnitude margin and its contribution decreases continuously through the transition. PIM controls the high-magnitude center. For unavailable or product-invalid cells, the validity override sets the effective InSAR weight to zero, preventing a low PIM magnitude from admitting an unsupported radar value.

PIM and fusion have the same calculation-grid minima, −1116.3 and −1564.9 mm, because the high-magnitude centers use PIM alone. The first field is an interval increment; the second is cumulative and may contain both lower-slice deformation and residual compaction. The calculation grids and their field definitions are provided in Supplementary Data S1.

### 3.4. Reconstruction Agreement Assessment

**Figure 11:**
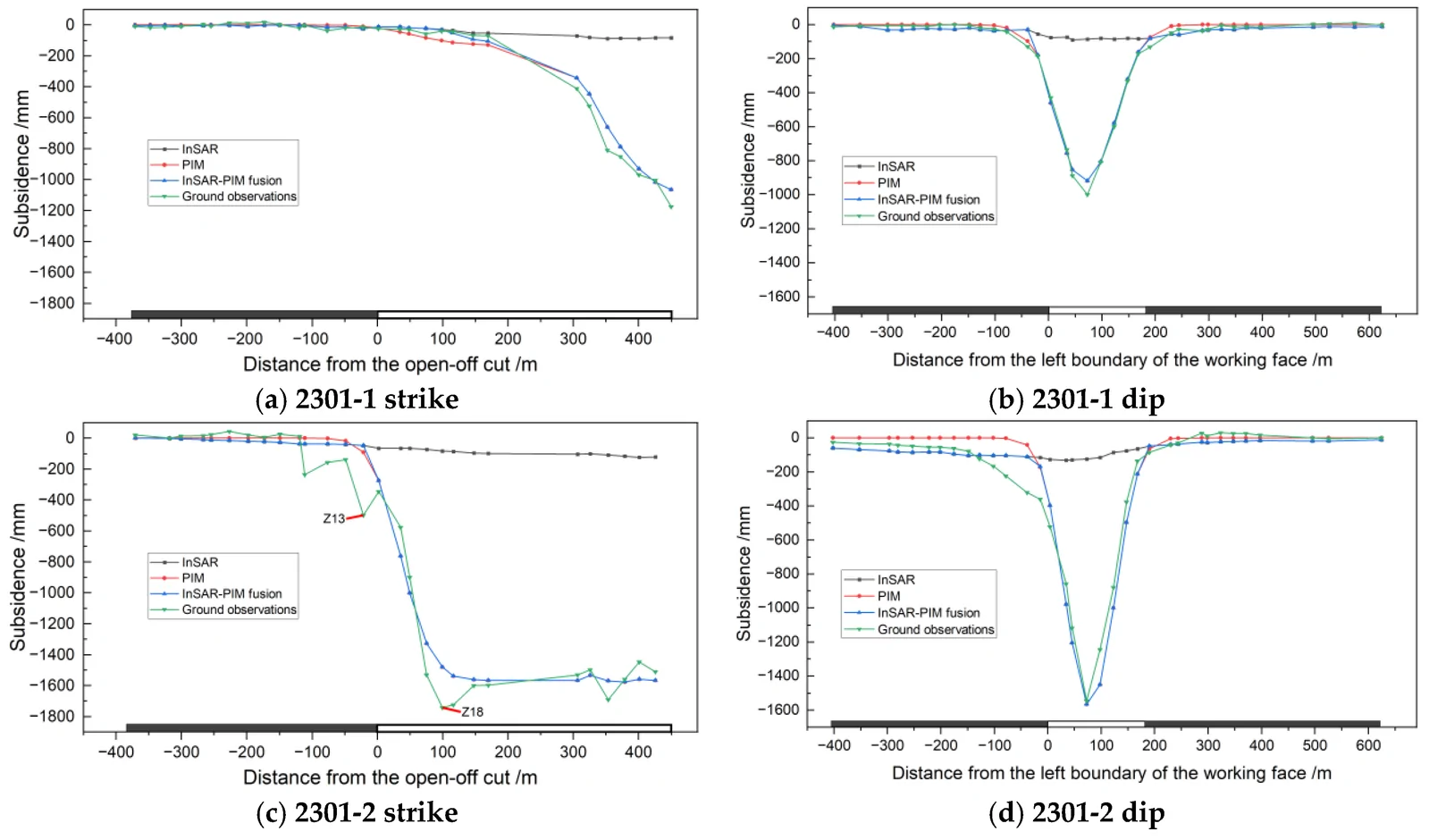
InSAR, field-only PIM, dynamic fusion, and ground observations (leveling or RTK). (**a**,**b**) 2301-1: interpolated 16 July 2015 start to 23/28 May 2016 InSAR/field endpoints. (**c**,**d**) 2301-2: 30 June/6 July 2018 InSAR/field starts to 25/21 February 2019 endpoints. Z13 and Z18 are labeled at their ground-observation positions in panel (**c**).

Figure 11 compares InSAR, PIM, and fusion at the original field-observation locations. Stage 1 uses the 28 May 2016 field survey minus the interpolated 16 July 2015 start, whereas *D*-InSAR ends on 23 May. Stage 2 compares 21 February field observations with the 25 February SBAS field and retains the six-day baseline difference. The four profiles contain 30, 35, 28, and 34 real monuments.

InSAR underestimates the central trough, whereas PIM recovers much of its magnitude. Fusion retains the radar observations near product-valid margins and converges to PIM in the high-magnitude center. The measured peak magnitudes are 1175.4 and 1743 mm. The stage-1 model permits unequal dip-side offsets. The measured 2301-2 dip profile shows more pronounced subsidence on the left flank adjoining 2303 than on the opposite side; a comparable one-sided enhancement is not evident in the representative 2301-1 profile (Figure 11b,d). PIM and fusion underestimate subsidence along part of this stage-2 flank. Fusion does not reduce the PIM error on every profile; its transition behavior is evaluated separately in Section 3.5.

MAE, RMSE, maximum absolute error, and R^2^ were calculated at the representative field points (Table 8). Because those monuments also contributed to the full-data inversion, these statistics measure internal reconstruction agreement. The spatial-block results are reported separately in Section 3.5.

Full-data fusion RMSEs were 28.58–134.78 mm (R^2^ = 0.956–0.990). Relative to PIM, the fusion RMSE changes (fusion minus PIM) for the four profiles are −5.15, +4.37, +1.67, −17.54 mm. The two largest stage-2 strike fusion residuals are 448.39 mm at −21.50 m (Z13) and 263.40 mm at 99.35 m (Z18).

On the 2301-2 strike profile, MAE increased from 91.57 mm for PIM to 94.78 mm for fusion. At Z13, absolute error increased from 407.31 to 448.39 mm. At Z18, PIM and fusion both have an absolute error of 263.40 mm, so this point contributes to the large overall residual but not to the MAE difference between methods. Z13 accounts for 1.47 mm of the 3.22 mm profile-mean increase, with the remaining 1.75 mm arising from the other monuments. The two labeled points therefore illustrate large residuals, not a two-point explanation of the fusion penalty.

The profile-dependent RMSE differences indicate that fusion does not uniformly improve PIM accuracy. Section 3.5 examines its transition behavior separately.

### 3.5. Fusion Benchmarks, Parameter Robustness, and Spatial-Block Assessment

**Figure 12:**
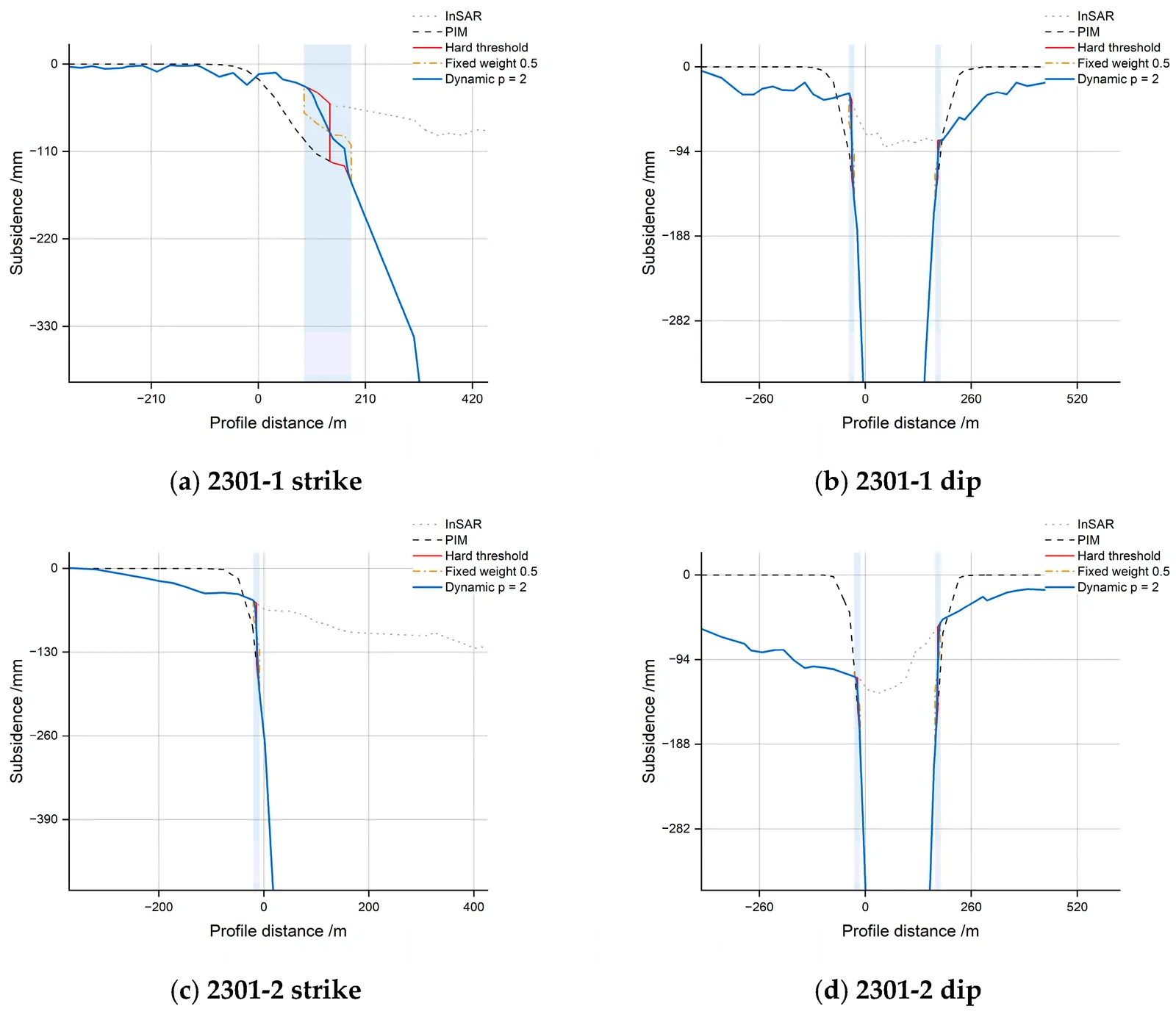
Transition-zone benchmark for hard-threshold stitching, fixed 0.5 weighting, and dynamic weighting (*p* = 2). Shaded bands denote *T*_min_ ≤ |*W*_PIM_| ≤ *T*_max_; the vertical axis is restricted to the transition range to reveal splice behavior.

Figure 12 compares hard-threshold stitching, fixed 0.5 weighting, and dynamic weighting on a 0.25 m profile grid. Continuity was evaluated on the dense grid, whereas accuracy was evaluated at the original measurement locations. Hard-threshold stitching introduces visible steps where the source changes. Fixed weighting reduces but does not remove the endpoint jumps, whereas dynamic weighting remains continuous at *T*_min_ and *T*_max_. Table 9 summarizes the accuracy and continuity metrics for the three fusion schemes.

Pointwise RMSE rankings differ among profiles, and the three schemes coincide where no original points sample their differing transition weights. The maximum theoretical splice jumps across the four profiles were 72.32–94.81 mm for hard stitching and 46.99–68.19 mm for fixed weighting, compared with zero for dynamic weighting. Maximum local gradients were 289.78–386.01, 189.13–278.84, and 1.71–21.47 mm m^−1^, respectively. These gradients describe interpolated-profile behavior at a fixed 0.25 m sampling interval; they are not new field measurements or a resolution-independent accuracy metric. Table 10 summarizes the sensitivity of fusion to the power parameter *p*.

Across *p* = 1–3, the largest within-profile RMSE change was 0.81 mm. The lowest RMSE was not consistently obtained at *p* = 2. The maximum local-gradient diagnostic ranged from 1.71 to 29.15 mm m^−1^ across all profiles and tested powers. We retained *p* = 2 as the stated moderate nonlinear transition, not as an empirically established optimum.

Supplementary Table S1 reports both RMSE and the coefficient of determination for all four tested powers on each original observation profile. These are full-data agreement statistics, with the same sparse transition sampling described below, rather than an independent selection test for the weighting exponent.

The stage thresholds were then varied using the nine configurations: base; ±10% *T*_min_; ±10% *T*_max_; both thresholds ±10%; a widened interval; and a narrowed interval. All configurations preserved 0 < *T*_min_ < *T*_max_. The baseline thresholds were retained after the sensitivity analysis. The results of the threshold sensitivity analysis are summarized in Table 11.

Across the nine threshold configurations, the combined-profile ranges were 2301-1: RMSE 35.91–37.21 mm and R^2^ 0.9866–0.9875; 2301-2: RMSE 109.80–110.33 mm and R^2^ 0.9683–0.9686. Few original monuments fall within the narrow transition intervals. These ranges therefore assess sensitivity of pointwise agreement; the continuity comparison uses the separately identified dense-profile diagnostic. Under the baseline thresholds, the four profiles contain 4, 1, 0, and 1 original transition points, respectively. Table 12 summarizes the same-site spatial-block fivefold assessment results after model selection.

Profile fusion RMSEs in the spatial-block test were 31.11–200.45 mm (R^2^ = 0.890–0.988). Stage-combined fusion RMSE/R^2^ were 62.16 mm/0.963 for 2301-1 and 166.79 mm/0.927 for 2301-2. The corresponding PIM RMSEs were 61.39 and 173.05 mm, so fusion increased RMSE by 1.25% in 2301-1 and reduced it by 3.62% in 2301-2. Relative to InSAR-only RMSEs of 332.08 and 712.51 mm, the reductions were 81.3% and 76.6%. Using each fold’s PIM prediction to classify its withheld points, none of the 65 stage-1 points and only 3 of the 62 stage-2 points fell within the transition interval. The overall spatial scores therefore provide little evidence for distinguishing transition-weight accuracy. Supplementary Data S1 reports this post hoc regional diagnostic; it was not used to retune the model or thresholds and is not a new independent validation. Because the same folds informed model selection, the spatial scores remain same-site post-selection estimates.

## 4. Discussion

### 4.1. Field-Constrained Inversion and Parameter Interpretation

The primary field-only fits recover the central subsidence pattern, while adding the 15 perimeter observations changes their own RMSE by only 0.03 and 0.01 mm (Table 7). Consequently, joint inversion is treated as a secondary comparison rather than the main source of improvement. Published layered- and repeated-mining studies show stage-dependent subsidence responses [34,35], while dynamic-subsidence and basin-margin studies provide methodological context [36,37]. These studies do not independently validate the present parameters.

The selected *q*/tan*β* values are 0.900/3.101 for 2301-1 and 0.884/4.242 for 2301-2. Published comparators include *q* = 0.79 ± 0.10 and tan*β* = 2.4 from mine-provided empirical formulas for a mountainous area [38], q/tanβ = 0.49/2.64 and 0.95/2.53 for initial and repeated mining in a Loess Plateau case [39], and tanβ = 2.51–3.40 in high-intensity-mining cases [40]. The present stage-1 *q* is slightly above the first comparator range, and the stage-2 tan*β* is comparatively high. These values were obtained under different geological, geometric, and observation conditions and are not universal admissibility limits. In this study *q* does not increase between stages, so the parameter comparison cannot be used as evidence of repeated-mining amplification.

The stages also differ in mining thickness, extracted dimensions, temporal reference and subsidence development. The influence radius is fitted directly, whereas its conversion to tan*β* depends on the representative depth. These geometric and normalization differences can contribute to the apparent parameter contrast. The stage-2 flank asymmetry is consistent with an influence from 2303, potentially involving residual compaction or renewed deformation of previously disturbed overburden during lower-slice extraction. These contributions are not separately resolved by the effective reconstruction parameters. The present fits do not isolate a causal effect of repeated mining on the parameters.

Despite numerical convergence, spatial sensitivity remains appreciable: the stage-1 fold estimates of *q* range from 0.876 to 0.961, and the stage-2 estimates from 0.727 to 1.001. These ranges are not confidence intervals. In addition, the exact *q*–k scaling equivalence means that changing the terminal normalization can change *q* without changing the displacement curves. The estimates should therefore be interpreted as conditional reconstruction parameters, not transferable material constants.

The spatial-block bootstrap further indicates substantial conditional uncertainty (Supplementary Table S3). The central 95% percentile intervals for *q* were 0.801–1.439 for 2301-1 and 0.457–2.000 for 2301-2; the corresponding tan*β* intervals were 2.390–3.370 and 2.581–5.369. At least one fitted parameter reached a numerical bound in 14.4% and 6.6% of resamples, respectively, and the upper stage-2 *q* percentile was limited by the imposed bound. With only five blocks per profile and fixed geometry, model choice and normalization, these intervals are exploratory conditional estimates rather than calibrated unconditional confidence limits. Their breadth and overlap do not support a statistically resolved causal contrast between the stages.

### 4.2. Dynamic Weighting and Basin-Margin Transition

Figure 12 and Table 9 compare the three weighting schemes directly. Dynamic weighting does not give the lowest RMSE for every profile, but its value and first derivative match the outer weight branches at both thresholds for *p* = 2. This endpoint property does not imply that the fused field is globally smooth: source gradients and availability-mask boundaries remain relevant. Table 10 shows limited pointwise sensitivity to *p*, and Table 11 shows small changes in combined RMSE and R^2^ under ±10% threshold perturbations. The sparse transition sampling reported in Section 3.5 limits both the sensitivity tests and the spatial assessment as evidence of transition-zone predictive accuracy. The zero theoretical splice jump and dense-profile gradients instead characterize the transition under the stated interpolation and sampling scheme.

The empirical retrieval limits (approximately 149 mm for 2301-1 and 194 mm for 2301-2) are specific to the conditions of each stage and do not represent an intrinsic performance difference between D-InSAR and SBAS-InSAR. They depend on the interferometric strategy, image availability, processing parameters, and deformation characteristics of the two mining stages. Compared with sequential D-InSAR stacking, the multi-temporal network and time-series inversion of SBAS-InSAR improve observation robustness in marginal areas where coherence is preserved. The broader marginal deformation during the repeated-mining interval may reflect both lower-slice extraction and neighboring-goaf effects; it cannot be attributed to repeated mining alone.

### 4.3. LOS Projection and Topographic Sensitivity

At profile points where |*W*_PIM_| < *T*_max_, the estimated absolute vertical-projection biases from the reported profile-direction horizontal component have medians of 4.71, 38.41, 11.37, and 41.48 mm. The corresponding 95th percentiles are 18.08, 105.95, 92.69, and 129.98 mm, and maxima range from 26.31 to 152.49 mm. The larger dip-profile estimates show that horizontal motion can matter at the flanks. These calculations use one reported horizontal component per profile and are not a full-vector error bound. They are retained as sensitivity estimates rather than applied as a three-dimensional correction.

The projection used center-scene incidence angles rather than a pixelwise local-incidence-angle product. In the incidence-angle perturbation scenarios, decreasing or increasing either center-scene angle by 5° changes the projected vertical scale by −6.3% to +8.1%; the corresponding 10° perturbations change it by −11.3% to +18.7%. DEM-assisted geocoding improves geometric positioning, while quality filtering restricts the analysis to retained observations. These procedures do not recover displacement information lost through layover or shadow, nor do they remove slope-parallel motion. For the reproduced 2301-1 trend correction, excluding the goaf and a 300 m buffer was intended to reduce contamination by mining deformation; any loss of real low-frequency deformation remains unquantified. The 66,113 candidate stable pixels changed from +34.03 mm mean and 39.70 mm RMSE relative to zero before correction to −0.16 mm and 9.36 mm after correction. At the 15 selected stage-1 perimeter samples, the applied correction was 22.12–32.65 mm (mean 27.62 mm), so omitting it would materially change those inputs. Replacing the quadratic surface with a linear surface changed the profile corrections by an RMS of 8.85 mm (maximum 9.26 mm). The correction centered the background, but its effect on real topography-correlated deformation remains unquantified. The low-hilly terrain and the stable-area zero assumption remain sources of uncertainty.

### 4.4. Interpretation of the Negative InSAR R^2^ Values

The negative InSAR *R*^2^ values indicate poor agreement in the high-gradient center (Table 8). They occur when the InSAR residual sum of squares exceeds the total sum of squared deviations of the field observations from their mean. Product-valid margin observations can still be used selectively, but the projected InSAR field does not represent a complete vertical-subsidence solution.

The empirical retrieval limits are far below the field-observation peaks, so center-zone InSAR underestimation dominates the all-profile statistics. At product-valid margins, InSAR remains useful for locating the basin and representing local spatial variation. The fusion uses InSAR at product-valid margins and PIM where central observations are missing.

### 4.5. Method Limitations and Future Research Directions

The main limitations concern parameter identification, timing, and radar geometry. The three late campaigns and terminal-factor normalization are modeling choices; they do not establish ultimate subsidence. The same spatial folds were used for model selection and assessment, and the field observations lack complete pointwise precision metadata. PIM idealizes the overburden and terrain, while the thresholds require stage-specific calibration. The comparison includes the interpolated stage-1 start and the stated 4–6 day offsets. A single ascending geometry does not separate displacement components, and DEM geocoding does not explicitly correct local visibility or slope-parallel motion. Trend removal assumes stable ground and may absorb real low-frequency deformation. Finally, the 2016–2018 image gap and lack of dedicated 2303 residual-monitoring records prevent separation of lower-slice deformation, residual compaction, and neighboring-goaf effects. The observed flank asymmetry does not quantify any one contribution.

Future work should combine ascending and descending SAR with two-component GNSS, use higher-resolution terrain and pixelwise visibility modeling, and acquire independent validation campaigns. Multi-face, time-dependent PIM formulations with additional observations could help distinguish lower-slice deformation from upper- and neighboring-goaf contributions. Multi-site tests and prospectively fixed weighting/threshold rules are needed to evaluate transferability beyond the present case.

The reconstructed grids provide spatial coverage beyond the principal profiles, but observations along those profiles do not validate accuracy throughout the two-dimensional area. Likewise, continuity at magnitude thresholds does not establish smoothness across source-field discontinuities or InSAR availability-mask boundaries.

## 5. Conclusions

Coherence loss can remove reliable InSAR measurements from the high-gradient center of repeated-mining subsidence basins in low-hilly terrain. Field observations along the principal profiles, however, provide limited two-dimensional coverage at the basin margins. The proposed framework combines InSAR observations from the basin margins with PIM estimates in the center. It was applied to both mining stages of the 2301 working face to produce spatially complete model-assisted fields, with accuracy assessed along the principal profiles.

The main conclusions are as follows:

(1) D-InSAR and SBAS-InSAR were applied to the initial- and repeated-mining stages, respectively, according to image availability and processing conditions. Both methods retained usable observations near the basin margins. The stage-specific empirical effective retrieval limits were 149 mm and 194 mm, respectively, providing the upper thresholds used in the dynamic weighting function.
(2) Field-only multi-epoch fitting selected *q* = 0.900/0.884 and tan*β* = 3.101/4.242 for 2301-1/2301-2. The mean boundary offsets are 54.66/36.51 m; stage 1 uses unequal dip-side offsets, while stage 2 uses one common offset. The secondary InSAR-assisted fits differ little from these estimates. The parameters are conditional on the terminal normalization and are not interpreted as mechanism constants.
(3) Dynamic weighting (*p* = 2) removed the theoretical value jump at each splice and reduced dense-profile transition gradients relative to hard-threshold and fixed-weight fusion. Its pointwise accuracy was not better than PIM on every sparse profile. Pointwise agreement changed little over *p* = 1–3 and the nine ±10% threshold configurations, but sparse transition sampling limits what these tests establish about predictive accuracy.
(4) Stage-combined fusion RMSE/R^2^ were 62.16 mm/0.963 for 2301-1 and 166.79 mm/0.927 for 2301-2. Relative to PIM, RMSE increased by 1.25% in the initial-mining stage and decreased by 3.62% in the repeated-mining stage. Each spatial block was excluded from every campaign before refitting. These are same-site post-selection results because the folds also informed model selection; independent validation remains necessary.

The framework was developed for mining-subsidence monitoring in low-hilly terrain, with possible application in similar geological and topographic settings after site-specific validation. The approach could also be tested in other geophysical monitoring settings that combine remote-sensing observations with physical models.

## Figures and Tables

**Table 1:** Mining parameters of the study working face and adjacent working faces.

Working Face	Dip Length/m	Strike Length/m	Mining Thickness/m	Average Mining Depth/m	Average Coal Seam Dip/°	Mining Period
2301-1	176	996	2.6	390	5	2015.05–2016.01
2303	270	1356	5.2	390	5	2016.05–2017.05
2305	270	1356	5.2	390	5	2017.07–2018.07
2301-2	158	1021	2.4	390	5	2018.07–2019.01
2307	270	1356	5.2	390	5	2019.01–2019.12

**Table 2:** Interferometric pair parameters for the 2301-1 initial-mining stage.

Pair No.	Interferometric Pair Dates		Temporal Baseline/d	Perpendicular Baseline/m	Coherence Coefficient in the Subsidence Area
	Primary Image	Secondary Image			
1	16 July 2015	20150809	24	−141	0.41
2	9 August 2015	2 September 2015	24	69	0.48
3	2 September 2015	26 September 2015	24	23	0.54
4	26 September 2015	20 October 2015	24	−73	0.53
5	20 October 2015	13 November 2015	24	108	0.58
6	13 November 2015	7 December 2015	24	−56	0.57
7	7 December 2015	31 December 2015	24	−39	0.63
8	31 December 2015	17 February 2016	48	32	0.61
9	17 February 2016	12 March 2016	24	−6.9	0.63
10	12 March 2016	5 April 2016	24	−13.8	0.60
11	5 April 2016	23 May 2016	48	59.0	0.38

**Table 3:** Acquisition dates of Sentinel-1A images for the 2301-2 repeated-mining stage.

No.	Acquisition Date	No.	Acquisition Date	No.	Acquisition Date
1	30 June 2018	14	3 December 2018	27	8 May 2019
2	12 July 2018	15	15 December 2018	28	20 May 2019
3	24 July 2018	16	27 December 2018	29	1 June 2019
4	5 August 2018	17	8 January 2019	30	13 June 2019
5	17 August 2018	18	20 January 2019	31	25 June 2019
6	29 August 2018	19	1 February 2019	32	7 July 2019
7	10 September 2018	20	13 February 2019	33	19 July 2019
8	22 September 2018	21	25 February 2019	34	31 July 2019
9	4 October 2018	22	9 March 2019	35	12 August 2019
10	16 October 2018	23	21 March 2019	36	24 August 2019
11	28 October 2018	24	2 April 2019	37	5 September 2019
12	9 November 2018	25	14 April 2019	38	17 September 2019
13	21 November 2018	26	26 April 2019		

**Table 4:** Principal symbols used in the numerical PIM implementation.

Symbol	Meaning	Unit/Status
*W*(*x*, *y*)	Predicted vertical displacement; downward negative	mm
*W*_max_	Theoretical amplitude, 1000mq cosα	mm
m, *q*, *α*	Mining thickness, subsidence factor, seam dip	m; dimensionless; °
*H*	Reference mining depth used to report tan*β*	390 m
tan*β*, *r*	Major influence angle tangent; influence radius *H*/tan*β*	dimensionless; m
*k_t_*	Campaign development factor; terminal *k_T_* = 1	dimensionless
*L*, *D*	Recorded strike advance and panel width	m
*S*_1_–*S*_4_, *S*	Boundary offsets; *S* is their arithmetic mean	m
*x*, *y*	Local strike and dip coordinates	m

**Table 5:** Stage-specific threshold calibration for the weighting function.

Stage/Method	*T*_min_/mm	*T*_max_/mm	*p*	Operational Calibration
2301-1/D-InSAR	95	149	2	*T*_min_: six-pair high-coherence proxy; *T*_max_: observed corrected-product EERL
2301-2/SBAS-InSAR	103	194	2	*T*_min_: 95^th^ percentile of valid non-IDW output; *T*_max_: observed-product EERL

**Table 6:** PIM parameter sets used for the two mining stages.

Working Face	*q*	tan*β*	*r*/m	Mean *S*/m
2301-1	0.8998	3.101	125.75	54.66
2301-2	0.8839	4.242	91.93	36.51

**Table 7:** Field-only inversion and the secondary field-plus-InSAR comparison.

Stage	Inversion	*q*	tan*β*	Mean *S*/m	Theoretical *W*_max_/mm	Late-Window RMSE/mm	InSAR-Margin RMSE/mm
2301-1	Field + InSAR	0.8996	3.102	54.67	2330.0	50.23	42.57
2301-1	Field only	0.8998	3.101	54.66	2330.6	50.23	42.60
2301-2	Field + InSAR	0.8841	4.239	36.50	2113.8	119.07	40.45
2301-2	Field only	0.8839	4.242	36.51	2113.4	119.07	40.46

**Table 8:** Full-data internal agreement relative to the field-observation profiles.

Profile	Points	Method	MAE/mm	RMSE/mm	Max. Error/mm	R^2^
2301-1 strike	30	InSAR	181.79	375.70	1091.86	−0.088
		PIM	34.45	48.80	149.66	0.982
		Fusion	28.07	43.65	149.66	0.985
2301-1 dip	35	InSAR	141.08	289.52	913.60	−0.045
		PIM	17.27	24.22	80.56	0.993
		Fusion	20.31	28.58	98.78	0.990
2301-2 strike	28	InSAR	683.68	957.10	1660.09	−0.721
		PIM	91.57	133.11	407.31	0.967
		Fusion	94.78	134.78	448.39	0.966
2301-2 dip	34	InSAR	209.93	413.98	1421.26	−0.071
		PIM	74.28	101.66	280.33	0.935
		Fusion	62.80	84.13	211.22	0.956

**Table 9:** Accuracy and continuity comparison of the three fusion schemes. RMSE values are in mm. Local gradients are numerical diagnostics from profiles interpolated at 0.25 m spacing, not directly measured physical deformation gradients.

Method	RMSE *S*_1_-Strike	RMSE *S*_1_-Dip	RMSE *S*_2_-Strike	RMSE *S*_2_-Dip	Max. Splice Jump/mm	Max. Local Gradient/(mm m^−1^)
Hard threshold	45.36	28.60	134.78	83.81	72.32–94.81	289.78–386.01
Fixed 0.5	43.79	25.66	134.78	85.84	46.99–68.19	189.13–278.84
Dynamic *p* = 2	43.65	28.58	134.78	84.13	0	1.71–21.47

**Table 10:** Sensitivity of fusion to the power parameter *p*. RMSE values are in mm.

*p*	RMSE S_1_-Strike	RMSE S_1_-Dip	RMSE S_2_-Strike	RMSE S_2_-Dip	Maximum Local-Gradient Range/(mm m^−1^)
1.0	43.50	28.26	134.78	84.72	4.31–21.84
1.5	43.56	28.52	134.78	84.36	2.22–17.62
2.0	43.65	28.58	134.78	84.13	1.71–21.47
3.0	43.88	28.60	134.78	83.91	1.82–29.15

**Table 11:** Sensitivity to the stage-specific transition thresholds (nine configurations per stage).

Stage	Base *T*_min_/*T*_max_/mm	Combined RMSE range/mm	R^2^ Range	Transition Points	Max. Transition-Gradient Range/(mm m^−1^)
2301-1	95/149	35.91–37.21	0.9866–0.9875	3–6	12.82–27.40
2301-2	103/194	109.80–110.33	0.9683–0.9686	1–2	18.03–27.99

**Table 12:** Same-site contiguous spatial-block fivefold performance after model selection.

Profile	Fold Design	PIM RMSE/mm	PIM R^2^	Fusion RMSE/mm	Fusion R^2^
2301-1 strike	Spatial block	85.37	0.944	85.11	0.944
2301-1 dip	Spatial block	27.44	0.991	31.11	0.988
2301-2 strike	Spatial block	201.92	0.923	200.45	0.924
2301-2 dip	Spatial block	145.04	0.869	132.81	0.890

## Data Availability

Sentinel-1A data are available from the Alaska Satellite Facility (https://search.asf.alaska.edu/#/, accessed on 1 March 2024), precise orbits from the Copernicus Data Space Ecosystem (https://dataspace.copernicus.eu/, accessed on 1 March 2024), and SRTM data from USGS EarthExplorer (https://earthexplorer.usgs.gov/, accessed on 1 March 2024). Original mine-coordinate and survey records remain confidential and may be requested from the corresponding author subject to authorization. Supplementary Data S1 supplies local-coordinate analysis inputs and derived evidence for the revised fits and comparisons; it does not claim to replace the original survey precision records. The supplied archive does not include a complete regeneration chain for every auxiliary diagnostic, including extraction of horizontal movements from the original survey records; access to those records remains subject to authorization.

## Supplementary Materials

The following supporting information can be downloaded at: https://www.mdpi.com/article/10.3390/s26216706/s1, Supplementary Data S1 contains the local-coordinate field observations, source-height audit, full-precision parameters and constraints, campaign factors, model comparisons, spatial-block predictions, representative profiles, numerical grids, fusion benchmarks, power/threshold sensitivity, post hoc regional diagnostics of the existing spatial-block predictions, and scripts for the primary fits and principal numerical checks. Supplementary Information provides the power-sensitivity statistics (Table S1), general PIM symbol definitions (Table S2), conditional spatial-block bootstrap results (Table S3), and profile-azimuth information (Table S4). The archive also includes the bootstrap samples and scripts, pointwise horizontal-component calculations and the residual attribution for Figure 11c. It distinguishes calculation grids from display interpolation and retains the terminal-normalization and model-selection limitations.
