# Supplementary material for: Stage-Adapted InSAR–Probability Integral Method Fusion for Monitoring and Spatially Continuous Reconstruction of Repeated-Mining Subsidence
Source: Sensors (Basel). 2026 Oct 23;26(21):6706. doi: 10.3390/s26216706 (PMC13766067; doi:10.3390/s26216706)
Supplement: Supplementary file 1 [file sensors-26-06706-s001.zip › Supplementary_Data_S1/Supplementary_Information_Round2.pdf]

Supplementary information for sensors 4485067

Stage-Adapted InSAR–Probability Integral Method Fusion for Monitoring and Spatially Continuous Reconstruction of Repeated-Mining Subsidence

This supplement reports power-sensitivity statistics, symbol definitions, conditional parameter uncertainty and profile-direction information. The uncertainty analysis supplements the selected point estimates without changing the PIM fits used in the manuscript. The results support a conditional reconstruction interpretation, not a resolved causal contrast between mining stages.

S1 Weighting exponent sensitivity

The symmetric power-ratio rule was evaluated at the original field-observation locations with the manuscript thresholds and signed source displacements. The coefficient of determination uses the residual sum of squares relative to the within-profile observed sum of squares. These values measure full-data agreement, not independent prediction. The original transition-point counts are 4, 1, 0 and 1 for the four profiles; the largest within-profile RMSE difference across the tested powers is 0.81 mm.

Table S1. Full-data fusion agreement for the four tested weighting powers.

Stage	Profile	p	RMSE (mm)	R^2
2301-1	strike	1	43.50	0.985413
2301-1	strike	1.5	43.56	0.985371
2301-1	strike	2	43.65	0.985308
2301-1	strike	3	43.88	0.985160
2301-1	dip	1	28.26	0.990040
2301-1	dip	1.5	28.52	0.989855
2301-1	dip	2	28.58	0.989809
2301-1	dip	3	28.60	0.989796
2301-2	strike	1	134.78	0.965865
2301-2	strike	1.5	134.78	0.965865
2301-2	strike	2	134.78	0.965865
2301-2	strike	3	134.78	0.965865
2301-2	dip	1	84.72	0.955168
2301-2	dip	1.5	84.36	0.955547
2301-2	dip	2	84.13	0.955790
2301-2	dip	3	83.91	0.956021

S2 Symbols in the general PIM formulation

Equation (8) is the general two-profile formulation. Table 4 of the manuscript describes the reduced numerical implementation. The first general profile maps to dip and the second to strike; the numerical data instead use strike and dip coordinates labelled x and y. This distinction prevents the general-form symbols from being mistaken for additional independently fitted parameters.

Table S2. Symbols in Equation (8) and their relation to the numerical reduction.

Symbol	Definition
$W(x, y)$	Two-dimensional subsidence amplitude in the general formulation.
W_{max}	Theoretical full-extraction amplitude; the general expression uses metres when mining thickness is in metres.
m, q, α	Mining thickness, subsidence factor and coal-seam dip angle, respectively.
L_0, L_1	Recorded lengths assigned to the first and second general profiles before the opposing boundary offsets.
l, L	Effective first-profile length and angle-projected second-profile length in Equation (8). The symbol L in the numerical data instead denotes recorded strike advance before offsets.
S_1, S_2	Opposing offsets for the first general profile, mapped to the two dip boundaries in the numerical model.
S_3, S_4	Opposing offsets for the second general profile, mapped to the two strike boundaries in the numerical model.
θ	Propagation angle in the general length projection; distinct from the radar incidence angle. It is not independently inverted in the reduced model.
H_0, H_1, H_2	General-form depths for the first profile and the two sides of the second profile. Direction-specific depths are not fitted here.
$\tan\beta$	Tangent of the main influence angle; reported from the fitted influence radius using the representative depth of 390 m.
$W^0(x)$, $W^0(y)$	One-dimensional influence functions; the superscript zero labels these functions and is not a temporal observation epoch.
x, y	General profile distances referenced to the first effective boundary, not the raw numerical survey-coordinate origins.
erf	Gaussian error function.

In the implemented reduction, the effective dimensions are recorded panel dimensions less opposing offsets, the propagation-angle length projection is omitted, and a common influence radius is used. The positive metre-valued general profile product is converted to downward-negative millimetres. The four boundary offsets are retained in calculation; their arithmetic mean is reported only for compact tabulation.

S3 Conditional parameter uncertainty

For each stage, 500 bootstrap datasets were generated with random seed 20260914. Each surveyed profile was divided into five contiguous distance blocks. Five blocks were sampled with replacement within each profile, and the same multiplicity was applied to every campaign of each sampled monument. The original three-campaign spatial objective and complete-sequence factor estimation were retained. Spatial parameters and monotone campaign factors were refitted, while the selected model, recorded geometry, original bounds and terminal factor of one were fixed. Model selection was not repeated.

Three deterministic starts were used per resample, including the full-data solution. All 500 fits per stage converged. Fourteen representative resamples, including the smallest and largest subsidence-factor solutions in each stage, were additionally checked with 24 starts; objective differences were below one part per million. Numerical convergence does not establish parameter identification. Resamples reaching the original bounds were retained in the percentile summaries.

Table S3. Conditional spatial-block bootstrap percentile intervals.

Stage	Parameter	Point estimate	2.5th percentile	97.5th percentile
2301-1	q	0.8998	0.801	1.439
2301-1	r (m)	125.751	115.715	163.204
2301-1	$\tan\beta$	3.101	2.390	3.370
2301-1	Mean S (m)	54.659	43.120	67.139
2301-2	q	0.8839	0.457	2.000
2301-2	r (m)	91.933	72.639	151.121
2301-2	$\tan\beta$	4.242	2.581	5.369
2301-2	Mean S (m)	36.512	0.000	60.625

At least one fitted parameter reached a bound in 72 of 500 resamples (14.4%) for 2301-1 and 33 of 500 (6.6%) for 2301-2. The upper percentile of the stage-2 subsidence factor is truncated by the imposed upper bound of 2.000. All boundary-hit flags, full samples, offset intervals and conditional covariance matrices are supplied in bootstrap_results.json. The mean offset is derived from the fitted offset constraints, not treated as an additional free parameter.

These intervals characterize resampling variability conditional on the selected model and fixed inputs. With only five blocks per profile, they are exploratory and need not have nominal frequentist coverage. They do not incorporate uncertainty in geometry, the terminal normalization, block design or model selection. The broad, overlapping intervals do not demonstrate a statistically separated stage effect or support attributing parameter differences solely to repeated mining.

S4 Horizontal component projection and residual attribution

Profile-axis bearings were obtained from the dominant direction of the east–north survey coordinates, clockwise from survey-grid north and modulo 180°. The source reports specify Beijing 1954 coordinates, with Y east and X north. Table S4 gives the angles used under the approximate alignment of survey-grid north with the radar azimuth reference; no grid-to-geographic-north rotation was applied.

Table S4. Angles used in the componentwise horizontal-motion calculation.

Stage	Profile	Profile axis (°)	Radar look (°)	Incidence (°)
2301-1	strike	0.106	76.995	39.285
2301-1	dip	84.878	76.995	39.285
2301-2	strike	0.194	76.890	38.558
2301-2	dip	84.465	76.890	38.558

The absolute horizontal look-direction component and its contribution to vertical-projection bias were calculated as

$$|u_{\mathrm{look}}| = |u_{\mathrm{p}} \cos(\psi_{\mathrm{look}} - \alpha_{\mathrm{p}})|, \quad B_{\mathrm{v}} = |u_{\mathrm{look}}| \tan\theta_{\mathrm{inc}}$$

where u_{p} is the reported profile-direction horizontal movement, α_{p} is the profile-axis azimuth, ψ_{look} is the radar horizontal look azimuth, θ_{inc} is the incidence angle, and B_{v} is the magnitude of the vertical-projection bias contributed by that component. The original survey-frame orientation and any additional local rotation have not been independently verified; the conditional projection check below bounds only the projection-related convergence. Reversing the movement sign or the profile axis by 180° leaves these magnitudes unchanged; an arbitrary north-reference rotation does not. The reports define south-to-north movement as positive on the strike profiles, but the results here remain absolute single-component sensitivity estimates, not signed corrections or complete three-dimensional error bounds. The pointwise inputs and numerical check are supplied in horizontal_components_round2.csv and verify_round2.py.

The conditional north-reference check used 65 archived pairs of survey coordinates and converted longitude/latitude. A Gauss–Krüger projection on the Krassowsky 1940 ellipsoid (semi-major axis 6378245 m; inverse flattening 298.3), with central meridian 112.5° E, latitude of origin 0°, scale factor 1, false easting 500000 m and false northing 0 m, reproduced these pairs with a planar RMS discrepancy of 0.295 m and a maximum of 1.094 m. No datum transformation or additional local rotation was applied. Because the longitude/latitude values were themselves obtained by conversion, these discrepancies measure coordinate-pair consistency, not independent positioning accuracy or a verified Beijing 1954-to-WGS84 transformation.

Under these projection assumptions, grid convergence at the archived points ranged from −0.00179° to +0.00482°. Applying this range as an exploratory angular perturbation to the 127 existing horizontal-component inputs changed the estimated absolute vertical-projection bias by at most 0.086 mm. This is a sensitivity scenario based on the archived stage-1 coordinate range, not a verified pointwise correction for both stages or a bound on any additional survey-frame rotation. The published profile azimuths and baseline projection statistics are unchanged.

Supplementary Data S1 includes the aggregate diagnostic and a script reproducing the 127-input perturbation; reproducing the coordinate-pair check requires the original survey-coordinate records, subject to the stated access restrictions.

On the repeated-mining strike profile, PIM and fusion MAEs are 91.57 and 94.78 mm across 28 monuments. At Z13 (−21.50 m), the absolute errors are 407.31 and 448.39 mm; this accounts for 1.47 mm of the 3.22 mm profile-mean difference. At Z18 (99.35 m), both errors are 263.40 mm, so this point contributes nothing to the MAE difference. The other monuments together account for the remaining 1.75 mm. Figure 11c marks both ground-observation positions; the supplied Origin project retains the original curve data.
